# Biomarkers for Early Cancer Detection: A Landscape View of Recent Advancements, Spotlighting Pancreatic and Liver Cancers

**DOI:** 10.1021/acsptsci.3c00346

**Published:** 2024-02-14

**Authors:** Rumiana Tenchov, Aparna K. Sapra, Janet Sasso, Krittika Ralhan, Anusha Tummala, Norman Azoulay, Qiongqiong Angela Zhou

**Affiliations:** ∥ CAS, a division of the American Chemical Society, Columbus, Ohio 43210, United States; ‡ Excelra Knowledge Solutions Pvt. Ltd., Hyderabad-500039, India; § ACS International India Pvt. Ltd., Pune-411044, India

**Keywords:** biomarker, cancer, diagnosis, prognosis, detection, monitoring, pancreatic cancer, liver cancer

## Abstract

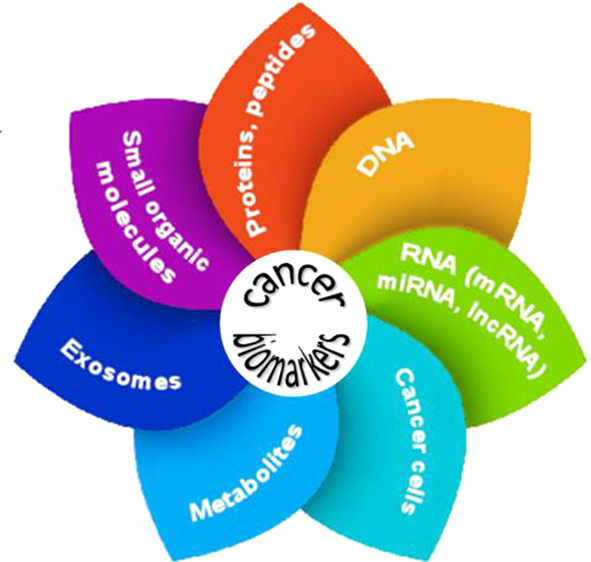

Cancer is one of the leading causes of death worldwide. Early cancer detection is critical because it can significantly improve treatment outcomes, thus saving lives, reducing suffering, and lessening psychological and economic burdens. Cancer biomarkers provide varied information about cancer, from early detection of malignancy to decisions on treatment and subsequent monitoring. A large variety of molecular, histologic, radiographic, or physiological entities or features are among the common types of cancer biomarkers. Sizeable recent methodological progress and insights have promoted significant developments in the field of early cancer detection biomarkers. Here we provide an overview of recent advances in the knowledge related to biomolecules and cellular entities used for early cancer detection. We examine data from the CAS Content Collection, the largest human-curated collection of published scientific information, as well as from the biomarker datasets at Excelra, and analyze the publication landscape of recent research. We also discuss the evolution of key concepts and cancer biomarkers development pipelines, with a particular focus on pancreatic and liver cancers, which are known to be remarkably difficult to detect early and to have particularly high morbidity and mortality. The objective of the paper is to provide a broad overview of the evolving landscape of current knowledge on cancer biomarkers and to outline challenges and evaluate growth opportunities, in order to further efforts in solving the problems that remain. The merit of this review stems from the extensive, wide-ranging coverage of the most up-to-date scientific information, allowing unique, unmatched breadth of landscape analysis and in-depth insights.

Cancer is a leading cause of death worldwide,^1^ with incidence of cancer expected to rise as a result of lifestyle deviations and a rapidly aging population.^2^ The occurrence of cancer increases dramatically with age, most likely due to the accumulation of risks for specific cancers, combined with the tendency for cellular repair mechanisms to become less efficient upon aging.^3^ Early detection is critical to reducing cancer morbidity and mortality.^4,5^

## Early Cancer Detection Rationale

Early cancer diagnosis is essential because it can save lives, improve treatment outcomes, reduce suffering, and lessen the economic and emotional burdens associated with cancer. Public awareness, screening programs, and research efforts are critical components of achieving early diagnosis and better cancer care. Specifically, early cancer diagnosis is crucial for certain essential reasons, and it forms the foundation of effective cancer care and treatment.^6−8^

One of the primary motivators for early cancer diagnosis is that treatment is often more effective when cancer is detected at an earlier, localized stage. At this stage, the tumor is typically smaller and has not spread to other parts of the body, making it more amenable to curative treatment options such as surgery, radiation therapy, or targeted therapy. Early cancer diagnosis is also associated with higher survival rates. When cancer is identified at an advanced stage, the chances of successful treatment and long-term survival significantly decrease. Early detection allows for timely intervention and a better chance of controlling or curing the disease.

Detecting cancer at an advanced stage often requires more aggressive and debilitating treatments, such as extensive surgeries and higher doses of chemotherapy or radiation therapy. Early diagnosis can lead to less invasive treatments with fewer side effects, resulting in a better quality of life for the patient. Treating cancer in its advanced stages is not only less effective but also more expensive. Late-stage cancer often requires prolonged hospitalization, multiple treatments, and supportive care, all of which contribute to higher healthcare costs. Early diagnosis can reduce the financial burden on patients and healthcare systems.

Cancer has the potential to metastasize, or spread, to other organs and tissues, which can make it much more challenging to treat and control. Early diagnosis and treatment can help prevent or minimize the spread of cancer, limiting its impact on the body. Also, when cancer is detected early, there is a better chance of preserving the normal function of affected organs or tissues. For example, in the case of breast cancer, early detection may allow for breast-conserving surgery (lumpectomy) rather than a full mastectomy.

Early cancer diagnosis often involves screening programs for individuals at high risk due to factors such as family history, age, genetic predisposition, or exposure to carcinogens. Identifying at-risk individuals and monitoring them regularly can lead to the detection of cancer at an earlier, more treatable stage. Early diagnosis can provide patients and their families with a sense of control and the opportunity to make informed decisions about treatment and lifestyle changes. It may also reduce anxiety and emotional distress associated with late-stage cancer diagnoses.

Finally, early cancer diagnosis contributes to the collection of data and samples that researchers can use to better understand cancer biology and develop new therapies and diagnostic methods. It fuels ongoing research efforts to improve cancer detection and treatment.

## Types of Biomarkers

Cancer biomarkers provide information about cancer and are therefore essential tools for early malignancy detection. Biomarkers are defined as a characteristic that is objectively measured and evaluated as an indicator of normal biological processes, pathogenic processes, or pharmacologic responses to a therapeutic intervention.^9^ A biomarker is any biomolecule, cellular structure, or bioactivity that can be measured and evaluated objectively as an indicator of pathogenic processes, normal biological processes, or pharmacological responses to a treatment.^10,11^ Various molecular, histologic, radiographic, or physiological entities or features are among the general types of biomarkers.^12^ Biomarkers can be classified based on their function, the way they are detected, or the kind of sample in which they are measured. Biomarkers have also been considered to include tools and technologies applied in the prediction, diagnosis, and pharmacological responses to a therapeutic treatment.^13^

**Figure 1:**
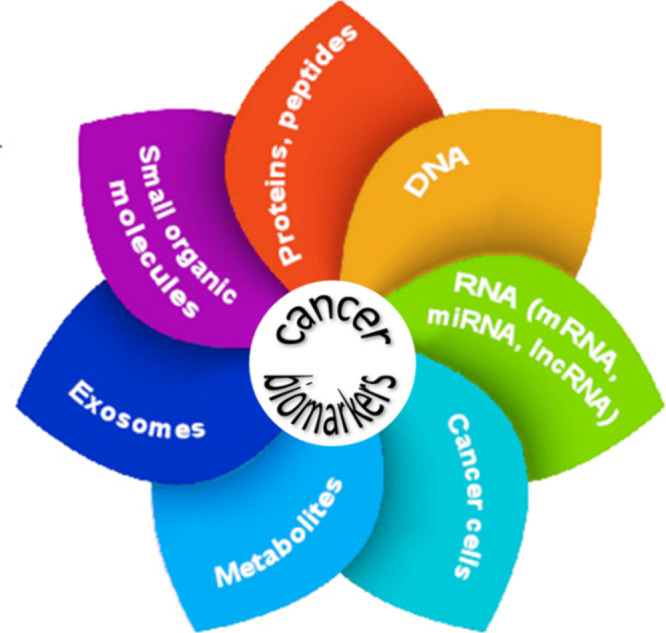
General types of cancer biomarkers.

Cancer biomarkers belong to a variety of biological molecule types, such as various proteins including enzymes, hormones and hormone receptors, tumor-associated antigens, serum and tissue proteins, etc.,^14^ as well as nucleic acids—DNA and RNAs including messenger RNAs (mRNAs), long non-coding RNAs (lncRNAs), and microRNAs (miRNAs),^15^—and exosomes,^16^ cellular metabolites, and various organic materials (Figure 1).^17,18^

- **Protein** biomarkers involve the measurement of body protein levels, their modifications, localization, or structural changes^19,20^ Examples include prostate-specific antigen (PSA) for prostate cancer and CA-125 for ovarian cancer. Neural protein deposits such as amyloid beta are often imaged to measure damage in neurodegenerative disorders.^21^ Similarly, the phosphorylation state of a protein can be impacted by kinase inhibition.
- **Genetic** biomarkers involve analyzing DNA or RNA for mutations, deletions, or other genetic alterations that may be associated with cancer.^19,22^ Examples include BRCA1 (BReast CAncer gene 1) and BRCA2 (BReast CAncer gene 2) mutations in breast and ovarian cancer, and mutations in EGFR common in lung cancer. Gene expression as well as their epigenetic modifications can serve as genetic biomarkers. Changes in DNA methylation patterns can be indicative of cancer, which rationalizes the use of **methylation biomarkers**. Hypermethylation of certain genes is associated with silencing tumor suppressor genes.^23,24^
- **Cell-free DNA (cfDNA)/circulating tumor DNA (ctDNA**) shed by cancer cells into the bloodstream can be analyzed for genetic mutations and alterations associated with specific cancers.^25−27^
- **MicroRNA (miRNA)** involved in gene regulation can be used as biomarkers since their expression profiles can be altered in cancer.^28,29^
- **Metabolites**—changes in metabolite profiles can indicate cancer-related alterations in metabolism, which rationalizes the use of metabolomic biomarkers.^30,31^
- **Glycans** are sugar molecules attached to proteins and lipids. Changes in glycosylation patterns on proteins and lipids can be associated with cancer and can serve as biomarkers.^32,33^
- **Exosomes**, small extracellular vesicles released by cells, can carry cancer-related molecules and are being investigated as potential biomarkers.^34,35^
- **Imaging biomarkers** for techniques like MRI, CT scans, and PET scans can reveal specific features associated with cancer, such as the size and location of tumors. PET scans use radioactive tracers to highlight areas with high metabolic activity, often indicating the presence of cancer. MRI can provide detailed images of soft tissues and is commonly used in cancer diagnosis.^36,37^
- **Serologic** markers can include antibodies and antigens that are detected in blood tests, such as the human papillomavirus (HPV) test for cervical cancer.^38−40^

Cancer biomarkers can also be functionally classified into various types. The categories of biomarkers defined by the FDA-NIH Biomarker Working Group according to their clinical usage include susceptibility and risk, diagnostic, monitoring, prognostic, predictive, pharmacodynamic and treatment response, and safety biomarkers.^9,12^

- **Diagnostic** (screening) biomarkers are used to detect and identify a given type of cancer in an individual. These markers are expected to have high specificity and sensitivity.^41^
- **Prognostic** biomarkers are used once the disease status has been established. These biomarkers are expected to predict the probable course of the disease including its recurrence, and they therefore have an important influence on the aggressiveness of therapy.^42^
- **Predictive** biomarkers serve to predict the response to a drug before treatment is started. This marker classifies the responsiveness of the individuals to a particular treatment. These biomarkers mainly arise from array-type experiments that make it possible to predict clinical outcome from the molecular characteristics of a patient’s tumor.^43^
- **Response** biomarkers are used to show that a biological response, potentially beneficial or harmful, has occurred in an individual who has been exposed to a medical product or an environmental agent.^44^
- **Monitoring** biomarkers are measured repeatedly for assessing the status of a disease or medical condition or for evidence of a medical product’s or an environmental agent’s effect.^45^
- **Susceptibility/risk** biomarkers indicate the potential for developing a disease or medical condition in an individual who does not currently have a clinically apparent disease or medical condition.^46^
- **Safety** biomarkers are measured before or after an exposure to a medical product or an environmental agent to indicate the chances or extent of toxicity as an adverse effect.^47^

The choice of biomarker(s) depends on the type of cancer being investigated and the specific diagnostic needs. Often, a combination of biomarkers and diagnostic methods is used to increase the accuracy of cancer diagnosis and staging. Additionally, ongoing research continues to uncover new biomarkers and refine existing ones, improving our ability to detect and diagnose cancer at earlier stages.

Researchers have made significant progress in developing valuable and effective detection techniques based on the specific recognition of cancer biomarkers. Multiple methods have been explored including enzyme-linked immunosorbent assay (ELISA),^48,49^ colorimetric assay,^50^ electrochemical assay,^51^ polymerase chain reaction (PCR),^52,53^ surface plasmon resonance (SPR), surface-enhanced Raman spectroscopy (SERS),^54^ fluorescence methods,^55^ and others.^56^ However, despite intense efforts, cancer biosensors are yet to achieve satisfactory clinical diagnostic standards.

## Timeline of the Cancer Biomarker Development

The first diagnostic biomarkers to be used for cancer testing and screening were primarily proteins such as alpha-fetoprotein (AFP) and PSA, which were discovered and developed for clinical application in the early stages of biomedical research. With the advances of biotechnology, biomarker research has been transformed and advanced to include circulating tumor DNA (ctDNA) such as mutated EGFR gene,^57^ various types of RNA, particularly miRNA,^58^ genes released during genetic modification and cell division,^59^ peptides and proteins including hormones, receptors, antibodies,^60^ also lipids and other metabolites,^61^ circulating tumor cells (CTCs),^62^ and extracellular vesicles such as exosomes.^63^

**Figure 2:**
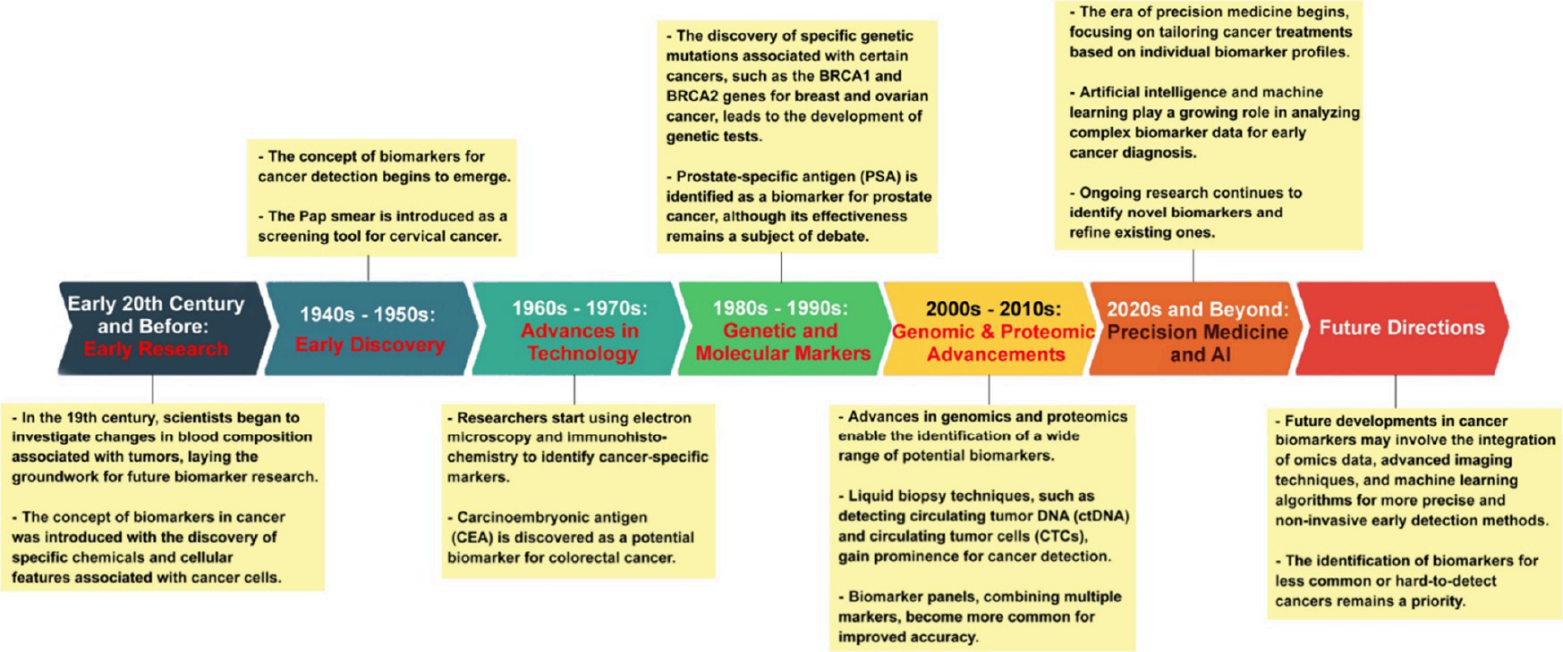
A concise timeline of key milestones and developments of biomarkers for early cancer diagnosis.^64−83^

The timeline for the development and discovery of biomarkers for early cancer diagnosis reveals a complex and ongoing process (Figure 2). It involves many years of research, clinical trials, and validation studies. In 1948, the existence of cell-free DNA (cfDNA) was first observed.^64^ cfDNAs are derived from necrotic and apoptotic cells, commonly released by all cell types. Further, numerous subsequent studies confirmed that tumor-specific patterns of alterations, such as chromosomal abnormality, somatic mutations, resistance mutation, aberrant methylation, and copy number variations, could be found in cfDNA, which can serve as potential targets for diagnosis of cancer through non-invasive approaches.^65,66^

The pursuit for non-invasive biomarkers appropriate for early cancer detection started in the 1970s and 1980s when certain so-called “cancer antigens” were discovered by introducing human cancer tissues into lab animals and testing the animal serum for antibodies against the human antigens in the extract. The first clinically applicable cancer biomarker identified this way in 1965 was carcinoembryonic antigen (CEA) in colon cancer tissue,^67,68^ and by the end of the 1970s, potential serum tests had been developed for a variety of cancers.^68^ Additional biomarkers developed in the 1980s included PSA, AFP, and cancer antigens 19-9 (CA19-9), 72-4 (CA72-4), 125 (CA125), and 15-3 (CA15-3).^69−74^

The development of monoclonal antibodies and immunoassays later on revolutionized biomarker research. This allowed for the detection of specific proteins associated with cancer. CEA became a widely used biomarker for monitoring cancer progression and treatment response. Advances in molecular biology and genetics led to the identification of genetic markers associated with inherited cancer syndromes. The discovery of BRCA1 and BRCA2 mutations in the 1990s marked a significant milestone in hereditary breast and ovarian cancer risk assessment.^75,76^

Genomic technologies like DNA microarrays and high-throughput sequencing that have emerged in the late 1990s to early 2000s, enabled the analysis of gene expression patterns in cancer cells.^77^ This period also saw the development of proteomics, which focused on identifying cancer-related proteins and peptides. The Human Genome Project^78^ and The Cancer Genome Atlas (TCGA) project^79^ provided valuable insights into the genetic mutations and alterations associated with various cancers. Liquid biopsy techniques began to gain attention for their potential to detect ctDNA and other biomarkers in blood samples, providing safe, accurate, non-invasive, and dynamic tracking of disease progression.^80^

Further, advances in single-cell sequencing allowed for a deeper understanding of tumor heterogeneity and the identification of rare cancer cells.^81^ Immune-related biomarkers, such as PD-L1 expression, gained prominence with the advent of immunotherapy for cancer treatment.^82,83^ The development of artificial intelligence (AI) and machine-learning (ML) algorithms accelerated the analysis of vast datasets for biomarker discovery. Ongoing research continues to focus on identifying novel early cancer biomarkers, especially those associated with rare cancers or those with poor prognosis. Efforts are made to develop minimally invasive or non-invasive methods for biomarker detection, such as through blood, urine, or breath tests. Multi-marker panels and composite biomarkers are being explored to improve diagnostic accuracy and reduce false positives.

Biomarkers must undergo rigorous validation and testing before receiving regulatory approval for clinical use. The timeline for regulatory approval and clinical adoption can vary, and biomarkers typically go through phases of clinical trials to demonstrate their clinical utility and safety. Development and adoption of cancer biomarkers vary by cancer type and the availability of technologies and funding for research. Advancements in molecular biology, genomics, and data analysis methods continue to shape the landscape of cancer biomarker discovery.

## Biomarker Research Growth

**Figure 3:**
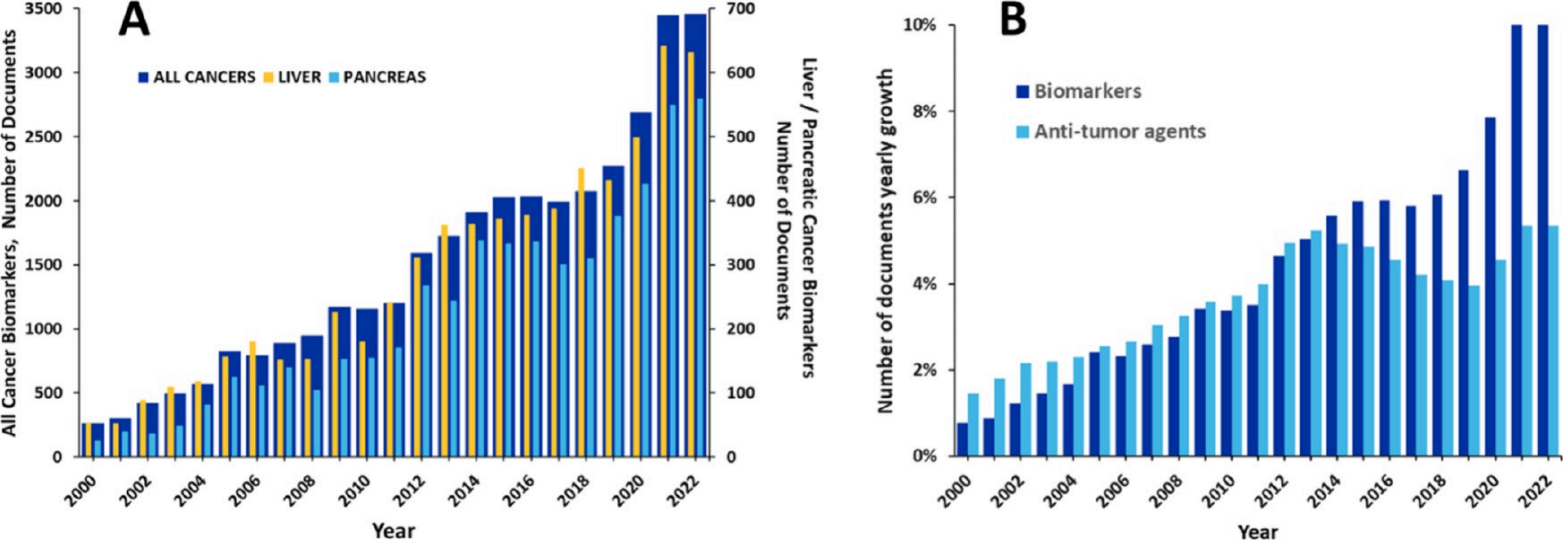
(A) Yearly growth of the number of documents (journal articles and patents) in the CAS Content Collection related to biomarkers for early cancer detection. (B) Yearly growth of the number of biomarkers vs anti-tumor agents-related documents.

In recent years, sizable methodological progress and a wealth of knowledge have promoted the advancement of the research on early cancer detection biomarkers, enhancing our understanding of its relationship to human physiology and pathologies. This is reflected in a persistent growth in the number of related scientific publications (journal articles and patents) in the recent decades (Figure 3A). A number of compendiums such as GOBIOM,^84^ GLOBOCAN 2020,^85^ OncoMX,^86,87^ MarkerDB,^88,89^ LiqBioer,^90,91^ BIONDA,^92,93^ BiomarkerBase,^94^ The Human Gene Mutation Database,^95^ Cancer Biomarkers database,^96^ CBD: a biomarker database for colorectal cancer,^97^ ONS Biomarker Database,^98^ and others^88^ have collected hundreds of proposed biomarker candidates. However, even though a large number of biomarker candidates have been proposed,^99^ very few have progressed to the stage of clinical validation along their clinical trial pathway.^100−104^

In Figure 3B, the yearly growth rate of the number of publications in the CAS Content Collection related to biomarkers for early cancer detection and those related to anti-tumor agents are compared. While initially the intense search for anti-tumor drugs resulted in higher growth rate in the area, in the past decade the growth rate in publications related to biomarkers for early cancer detection began to significantly dominate. This is a result of the insight that successful cancer treatment is only achievable at an early, localized stages of the disease, which requires efficient diagnostic strategies. This perception drew special attention to the early cancer detection and gave rise to the strong biomarkers research growth rate.

In this paper, we review the advances in the knowledge related to biomolecules and cellular structures used for cancer early detection, diagnosis, prognosis, and monitoring. We examine data from the CAS Content Collection,^105^ the largest human-curated collection of published scientific information, and analyze the publication landscape of recent research in order to provide insights into the scientific advances in the area. We also explore the Excelra Biomarker Insights datasets^106^ containing manually compiled information around biomarkers for selected disease indications. We discuss the evolution of key concepts in the field as well as the major technologies, the development pipelines of cancer biomarkers with a particular focus on pancreatic and liver cancer biomarkers. Pancreatic and liver cancers are known as some of the cancer types remarkably difficult to detect early, with particularly high morbidity and mortality,^107−110^ so we put a special emphasis on examining the advancements on the early detection of these two types of malignancies. The objective of the paper is to provide a broad overview of the evolving landscape of current knowledge on cancer biomarkers, to outline challenges and evaluate growth opportunities, in order to further efforts to solve the problems that remain. The novelty and merit of the article stem from the extensive, wide-ranging coverage of the most up-to-date scientific information accumulated in the explored databases, the CAS Content Collection and the Excelra Biomarker Insights datasets, allowing unique, unmatched breadth of landscape analysis and in-depth insights. We hope this report can serve as a useful resource for understanding the current state of knowledge in the field of cancer biomarker research and development.

### 1 Landscape View of the Cancer Biomarkers Research—Insights from the CAS Content Collection

**Figure 4:**
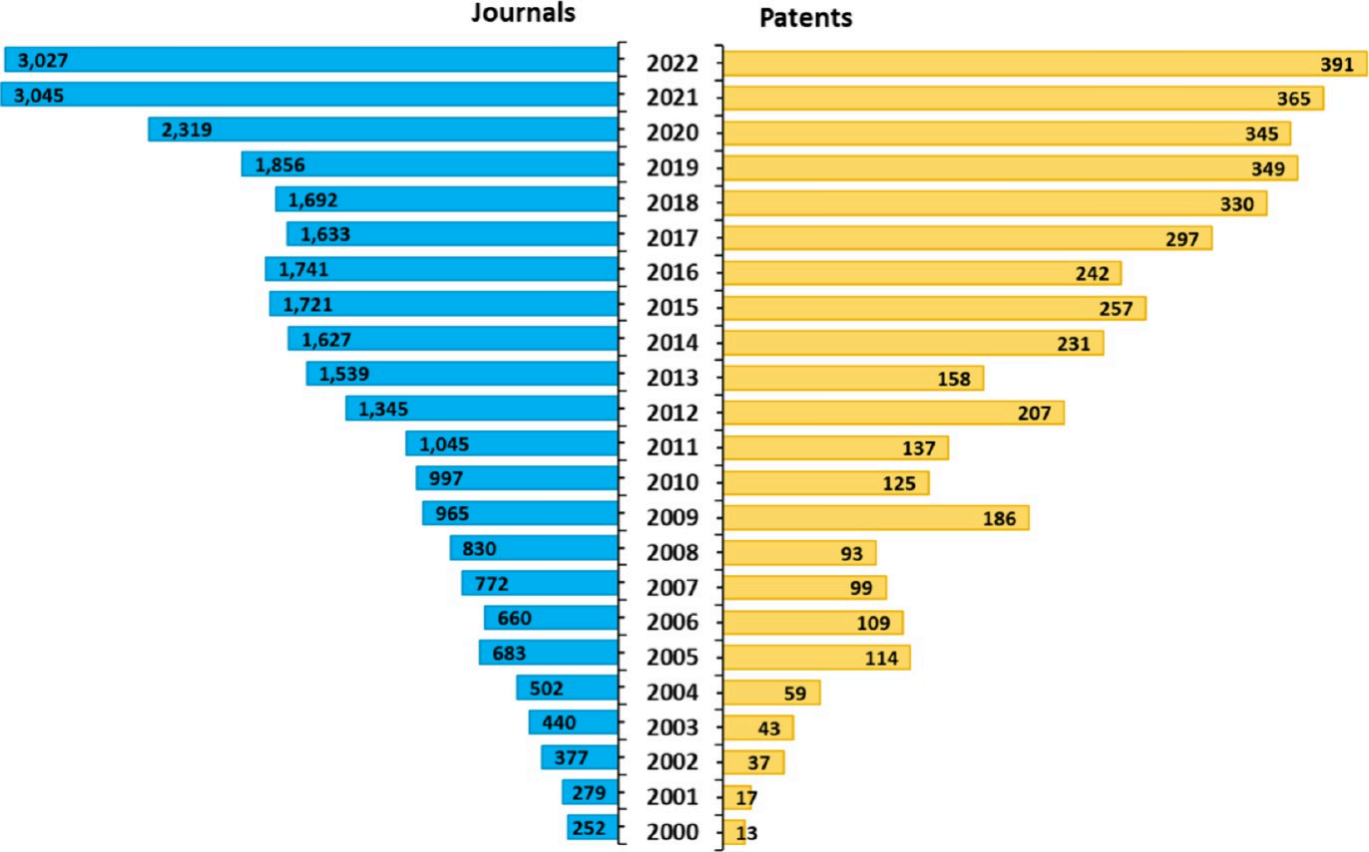
Yearly growth of the number of documents (journal articles, left, and patents, right) in the CAS Content Collection related to the research and development in the field of biomarkers for early cancer diagnosis.

The CAS Content Collection^105^ is the largest human-curated collection of published scientific information, which represents a valuable resource to access and keep up to date on the scientific literature all over the world across disciplines including chemistry, biomedical sciences, engineering, materials science, agricultural science, and many more, thus allowing quantitative analysis of global research publications across various parameters including time, geography, scientific area, medical application, disease, and chemical composition. Currently, there are over 30,000 scientific publications (mainly journal articles and patents) in the CAS Content Collection related to biomarkers for early detection and diagnosis of cancers. Of these, over 4,000 documents are related specifically to liver cancer, and over 3,000 documents to pancreatic cancer. There has been a steady growth of these documents (both journal articles and patents) over the past decades, with a nearly 30% increase in the number of journal articles in the past 2 years (Figure 4). The growth in the number of patents is still slower, indicating the stage of accumulation of scientific knowledge preceding its subsequent transfer into patentable applications.

**Figure 5:**
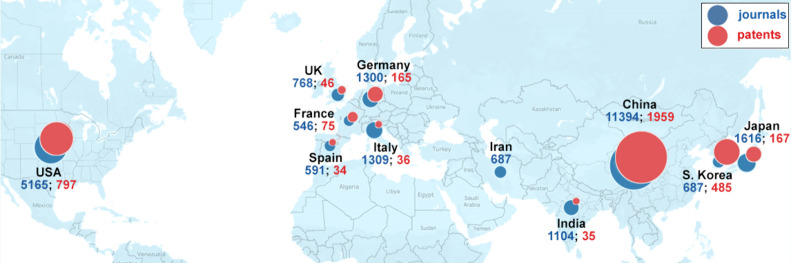
Top countries/regions with respect to the numbers of cancer biomarkers-related journal articles (blue) and patents (red).

**Figure 6:**
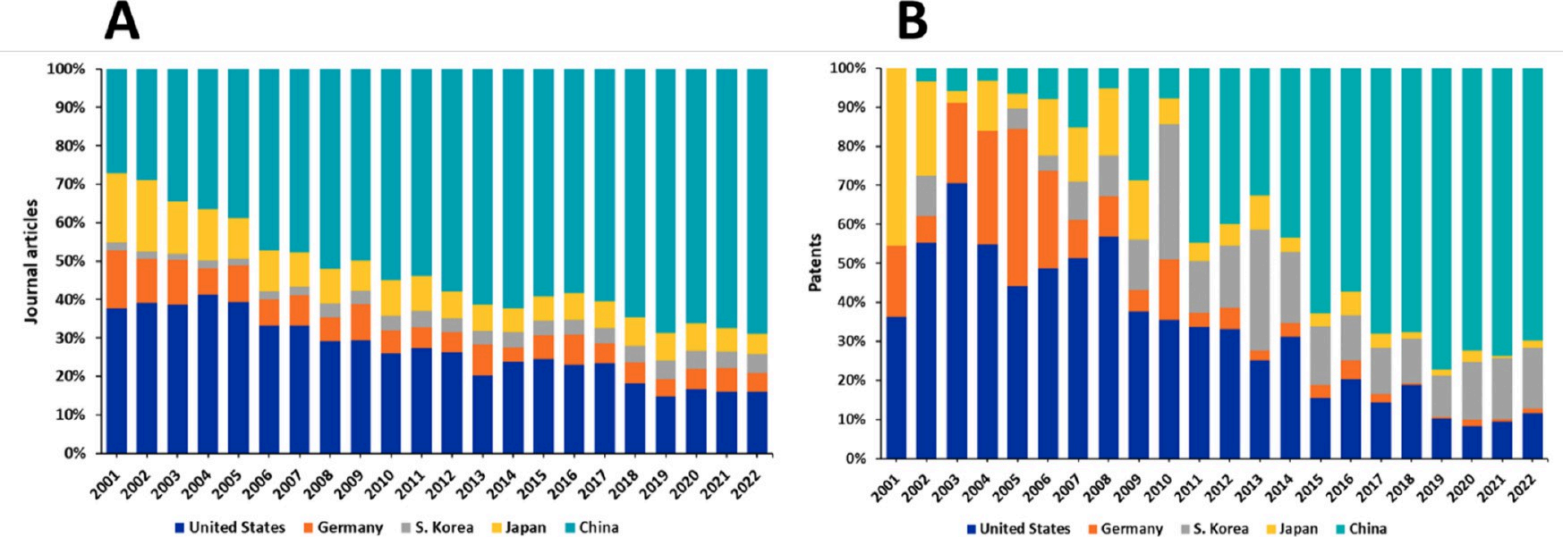
Annual contribution of the top countries/regions to the number of journal articles (A) and patents (B) related to the cancer biomarkers research.

China, the United States, South Korea, Japan, and Germany are the leaders with respect to the number of published journal articles and patents related to cancer biomarkers research, with China spotted as an eminent leader (Figure 5). A graph of the annual contribution of the top countries/regions to the number of journal articles and patents exhibits the gradual increase of the portion of publications from China and South Korea at the expense of those from the U.S., Japan, and Germany (Figure 6).

**Figure 7:**
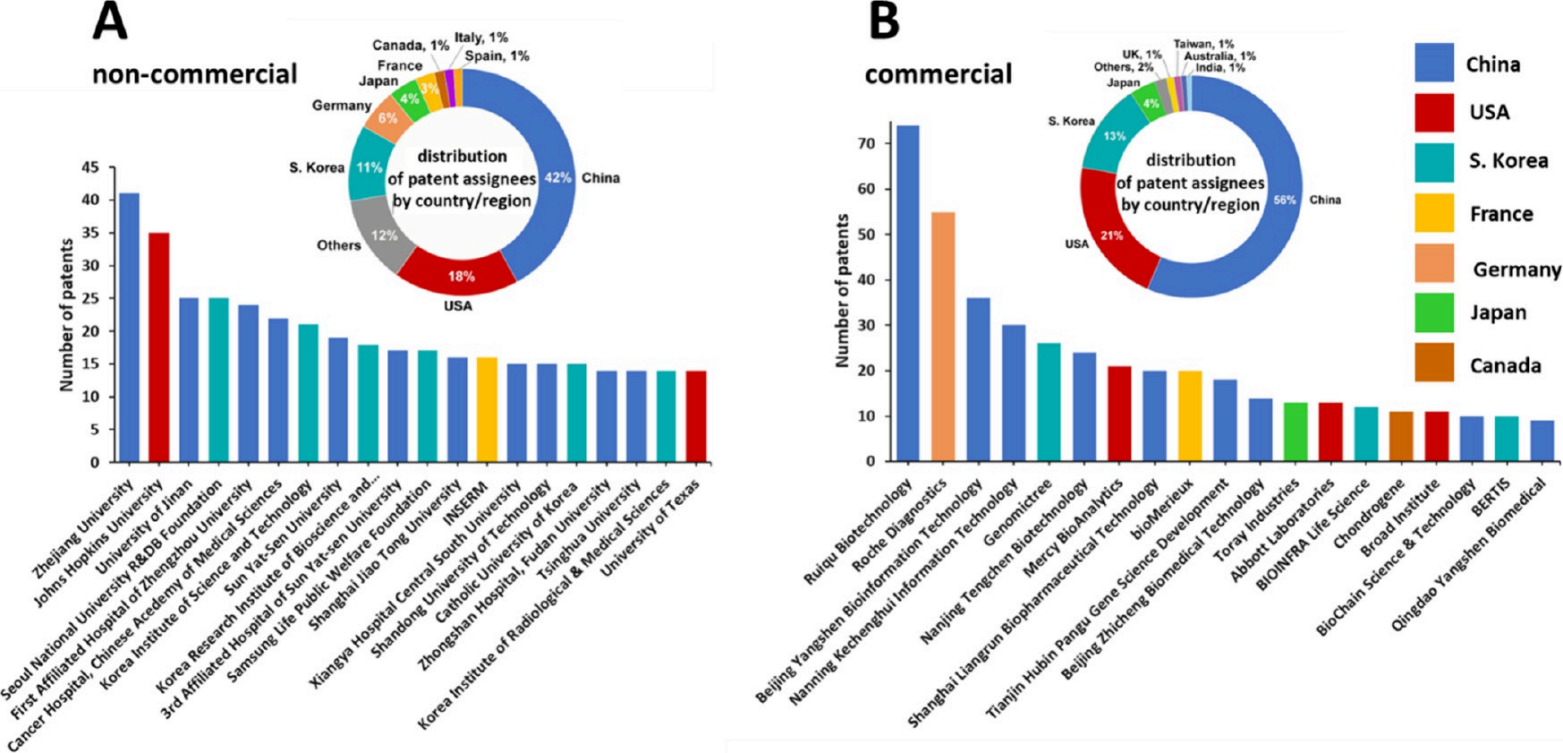
Distribution of patents between top non-commercial (A) and commercial (B) organizations. Donut charts indicate country-wise distribution of the top patent assignees, while bar graphs show breakdown of the top patent assignee organizations.

**Figure 8:**
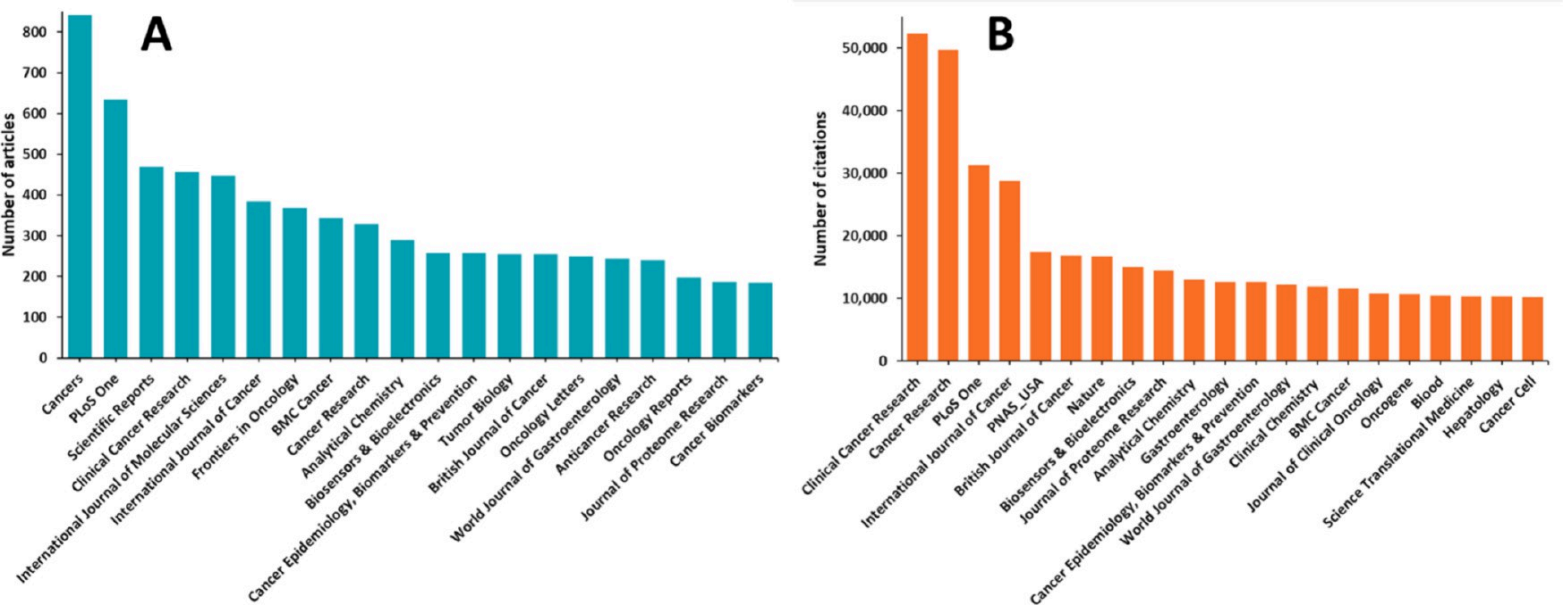
Top scientific journals with respect to the number of cancer biomarker-related articles published (A) and the citations they received (B).

The Ruiqu Biotech company, Zheijang University, and Johns Hopkins University have the largest number of patents (Figure 7). The journal *Cancers* publishes the highest number of articles related to cancer biomarkers (Figure 8A), while the journals *Clinical Cancer Research* and *Cancer Research* are the most-cited journals for cancer biomarkers research (Figure 8B).

**Figure 9:**
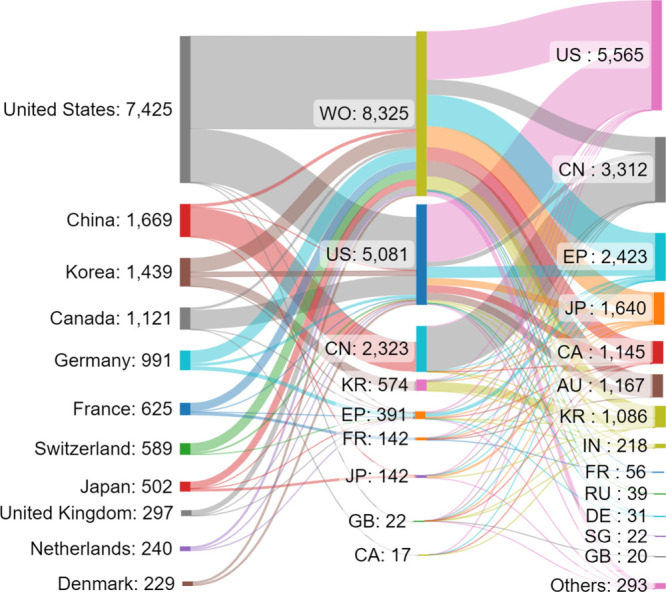
Sankey graph depicting the flow of cancer biomarker-related patents between assignee countries/regions (left), office where application is first filed (center). and final destination office (right). Only patents for which the entire flow information is available are included in the graph.

Patent protection is territorial and therefore the same invention can be filed for patent protection in several jurisdictions. We searched for all related filings pertaining to the cancer biomarkers. Certain patent family might be counted multiple times when they have been filed in multiple patent offices. Detailed analysis of the patent family data (Figure 9) indicating the complex flow of patents from patent assignee countries/regions or regions (left column) to the patent office wherein the application is first filed (center column) and the patent office where the application finally ends up (right column) is shown in Figure 9. There are diverse patent filing strategies: some patent assignees, such as those from China, file foremost in their home country patent office (CN), with a smaller proportion filing through the World International Patent Office WIPO (WO), or other jurisdictions. Others, such as the U.S.-based applicants, have a dominating number of WO filings. Most of the applicants tend to have a comparable number of filings in their home country and at WO, while also having a sizable number of filings at other patent offices such as the U.S. and European Patent Offices (US, EP), and others.

**Figure 10:**
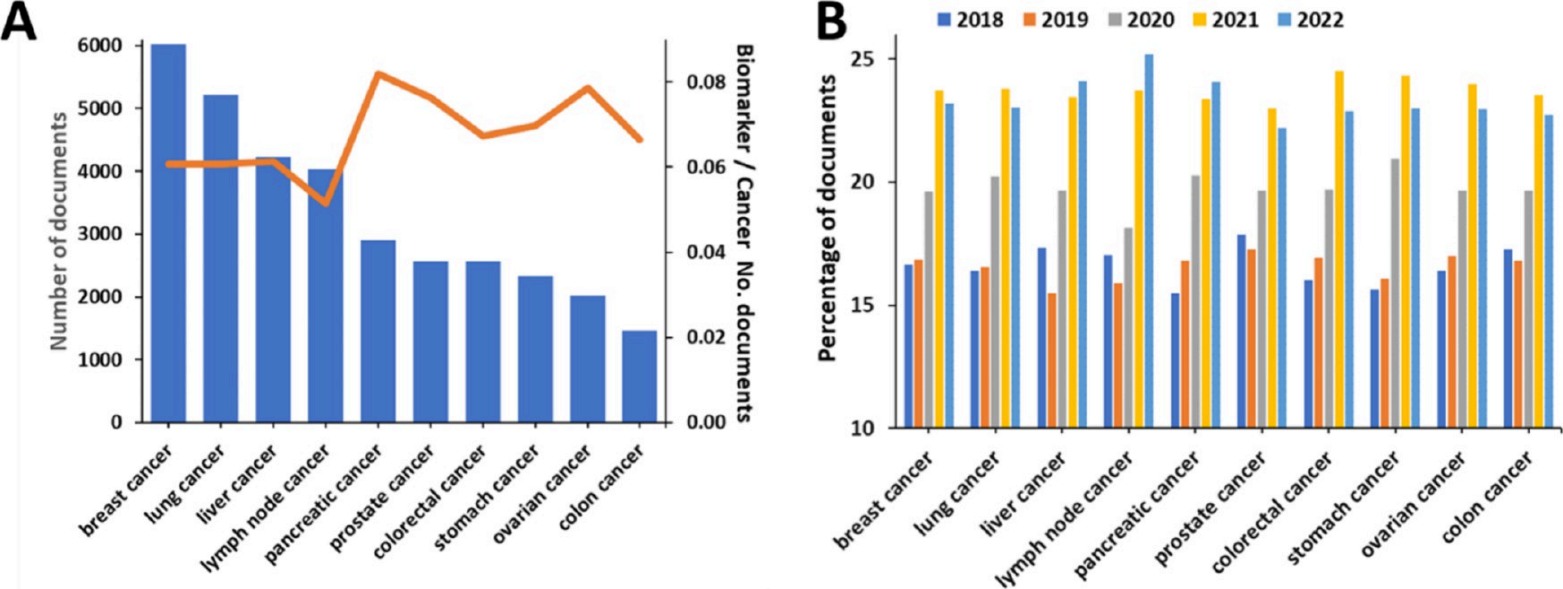
(A) Number of publications in the CAS Content Collection related to biomarkers for diagnosis of various cancer types (columns); the line indicates the ratio of biomarker to cancer documents. (B) Yearly growth of the biomarker-related documents for the past 5 years (2018–2022).

Breast cancer, lung cancer, and liver cancer are explored in the highest number of cancer biomarkers-related documents (Figure 10A). Noteworthy, when normalized over the all documents related to the specific cancer type, the value is the highest for the pancreatic cancer biomarkers, i.e., from all pancreatic cancer-related documents, the highest number are also associated with biomarkers, as compared to other cancer types (Figure 10A, orange line); this normalized number of documents value is similarly high for the ovarian cancer biomarkers (Figure 10A, orange line). With respect to annual growth, all major cancer types mark substantial growth in the recent 3-year period, but lymph node cancer, pancreatic cancer, and liver cancer are those drawing attention with an especially steady growth in the number of recent publications (Figure 10B).

**Figure 11:**
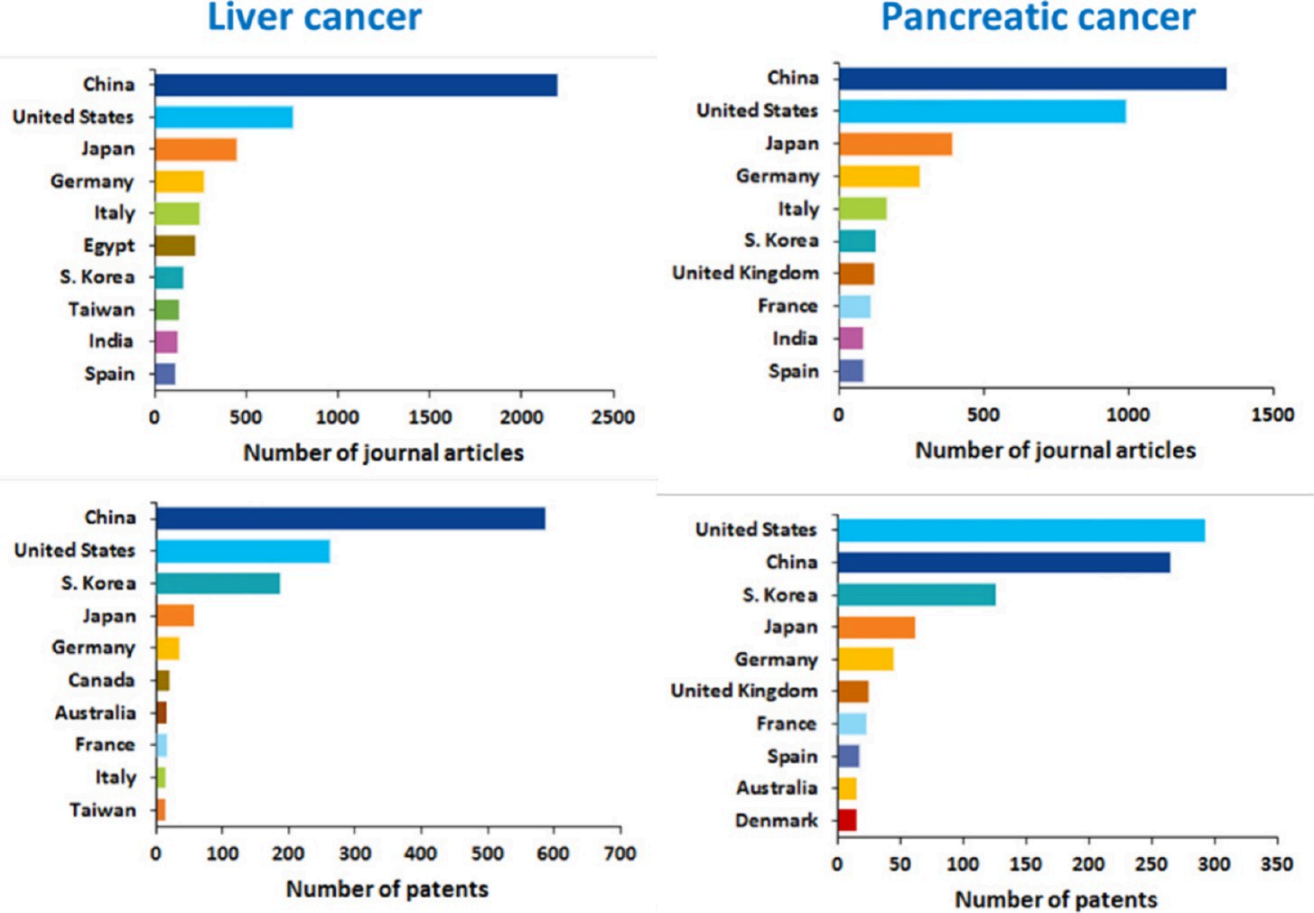
Top countries/regions with respect to the numbers of liver and pancreatic cancer biomarkers-related journal articles and patents.

China is the leader in both journal articles and patents related to liver cancer biomarkers, followed by the U.S., Japan, and South Korea (Figure 11, left). Regarding pancreatic cancer markers, China has the most journal articles, while the U.S. is leading in patents (Figure 11, right).

**Figure 12:**
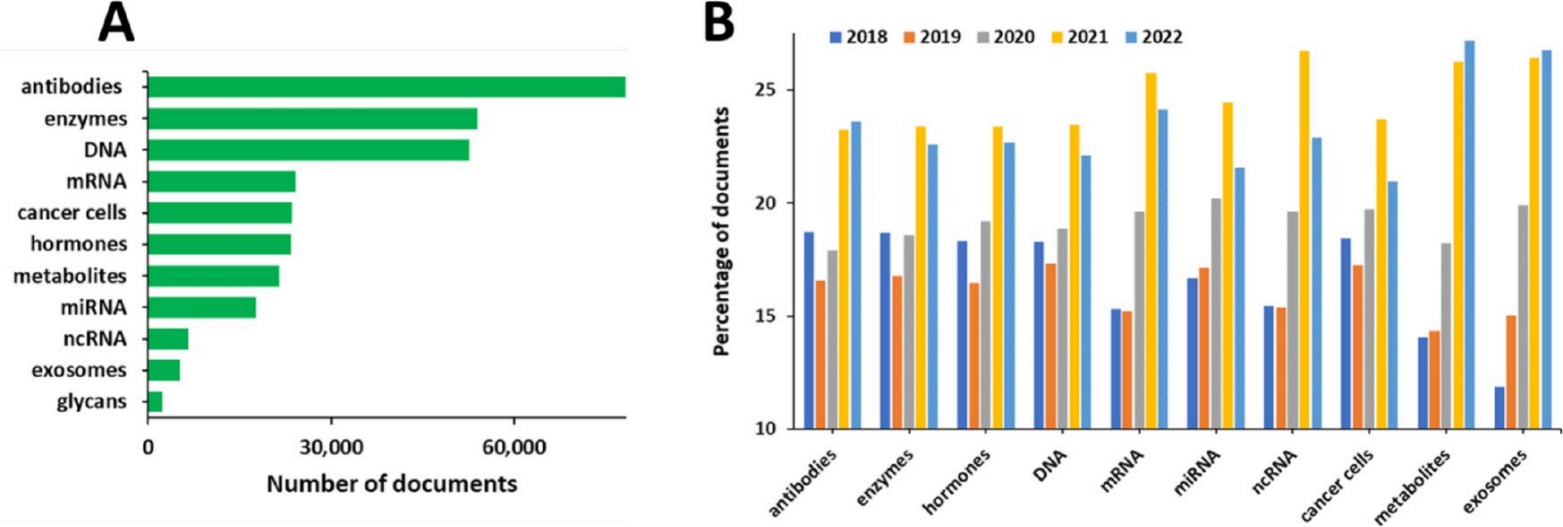
Number of publications in the CAS Content Collection related to various biomarker types for cancer diagnosis (A) and their yearly growth for the past 5 years (2018–2022) (B).

Cancer biomarkers belong to a variety of molecular types and structures including proteins, nucleic acids, cellular metabolites, also exosomes, and various other organic materials and tissues. Protein biomarkers have been among the first and the most widely used in cancer diagnostics. Most of them are based on cancer antibodies/immunoglobulins, enzymes, and hormones, as reflected by the number of published documents (Figure 12A). Protein biomarkers include overexpressed proteins (e.g., HER2), mutated proteins (e.g., p53), or proteins with tumor-specific post-translational modifications (e.g., KRAS mutations), which can be found in tumor tissue. Protein biomarkers detectable in blood or other body fluids also include tissue/cell-specific proteins that have enhanced levels in body fluids as compared to normal, e.g., PSA in the blood plasma of prostate cancer patients.^19^ Proteome characterization in cancer has been recently assessed by two initiatives, the Clinical Proteomic Tumor Analysis Consortium^111^ and the Human Protein Atlas.^112^

In recent years, metabolites^30,113^ and exosomes^16,114^ have emerged as promising new classes of markers and are exhibiting fast and consistent yearly growth in the number of published documents (Figure 12B). RNAs,^115^ specifically mRNA^116,117^ and ncRNA,^117,118^ are also among the attractive biomarker candidates, according to the yearly growth in their number of documents (Figure 12B).

There has been substantial progress in the field of biomarker detection technologies recently.^119^ Advanced biomarker detection methods have been explored and refined, including, for example, ELISA,^120,121^ gel electrophoresis,^122,123^ SPR,^124,125^ protein microarray,^126^ SERS,^127,128^ colorimetric tests,^129,130^ electrochemical analysis,^131,132^ and fluorescence methods.^133,134^ Each method has its own advantages and limitations. Thus, ELISA exhibits high sensitivity and specificity, allowing for the detection of low concentrations of biomarkers, but has limited multiplexing capability (ability to detect multiple biomarkers simultaneously); PCR-based methods are also of high sensitivity and provide quantitative information but are limited to nucleic acid biomarkers, are quite time-consuming, and may require complex instrumentation; mass spectrometry is of high specificity and has the ability to detect multiple biomarkers simultaneously, but exhibits limited sensitivity for some low-abundance biomarkers; next-generation sequencing allows for the analysis of multiple genes simultaneously, but is a costly and complex technology, and data analysis can be challenging; MRI provides structural information but is relatively expensive and not widely available in resource-limited settings; microarray technology allows high-throughput screening for multiple biomarkers, but has limited sensitivity for low-abundance biomarkers, etc.^119^ Along these lines, diagnostic platforms that would allow the detection of biomarkers at ultralow concentrations for the development and evaluation of novel biomarkers, and for early detection of cancer and treatment follow-up are needed.

**Figure 13:**
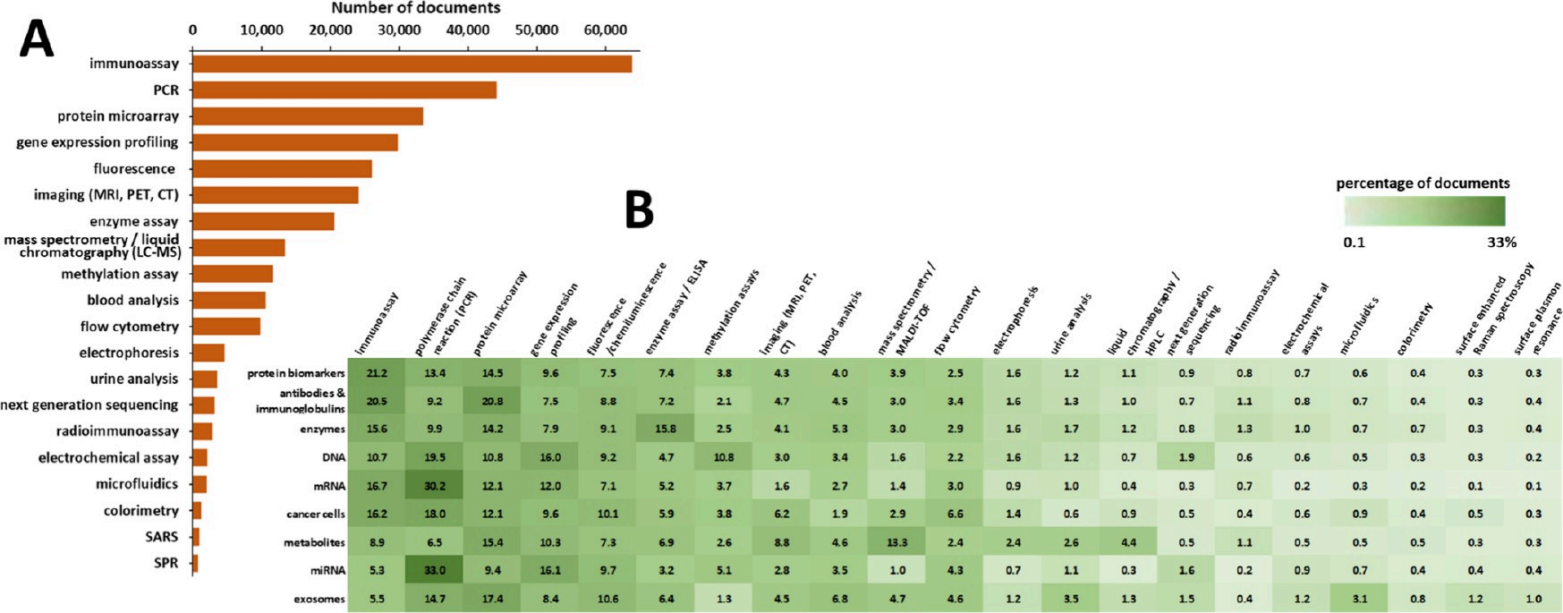
Number of publications in the CAS Content Collection related to various cancer biomarker detection methods (A) and a heatmap of their co-occurrence with the types of cancer biomarkers (B).

According to the CAS Content Collection data, immunoassays, PCR, protein microarrays, and gene expression profiling are the most widely used techniques for biomarker detection (Figure 13A). Indeed, immunoassays can provide a fast, simple and a cost-effective method of detection, with good sensitivity and specificity, automation options and versatility; PCR allows precise quantification of the biomarkers, with high reproducibility; microarray technology is appropriate for high-throughput screening for multiple biomarkers. PCR exhibits the highest co-occurrence with the various nucleic acid markers, particularly with mRNA and miRNA biomarkers (Figure 13B). Indeed, using PCR, RNA sequence traces can be amplified and thus RNA can be detected with high specificity and sensitivity.^135^ Protein biomarkers are mainly examined by immunoassays—proteins from tumor tissues are studied by immunohistochemistry, while ELISA is regularly used for body fluid protein biomarkers.^136^ In addition to PCR and gene expression profiling, methylation assays are commonly used for DNA markers analysis (Figure 13B). Cancer-specific DNA methylation in the cfDNA from the tumor derived blood samples is certainly a convenient strategy for minimally invasive detection and monitoring of cancer.^137^ Imaging, including magnetic resonance imaging (MRI), positron emission tomography (PET), computed tomography (CT), fluorescence assays, ultrasound imaging (ultrasonography), and others, has a strategic role in the management of cancer.^36,138,139^ Imaging biomarkers, which objectively inform on tumor biology, environment, as well as tumor changes in response to an intervention, notably complement genomic and molecular diagnostics. Next generation sequencing is the rising new DNA sequencing technology, offering ultrahigh throughput, scalability, and speed, and variant/mutation detection capability, revolutionizing genomic research.^140−142^ Mass spectrometry, which allow for the detection of many different metabolites, is between the most commonly used techniques for detection of metabolite biomarkers (Figure 13B).^143^ Extracellular vesicles such as exosomes are rich sources of circulating biomarkers detected in a variety of body fluids.^144,145^ Quantitative proteomics is the preferred technique for assessing exosome biomarkers (Figure 13B).

In recent studies, the CRISPR-Cas system has proven to be very effective early cancer diagnosis, along with many other fields of application.^146^ CRISPR-based genome and transcriptome engineering, and specifically CRISPR-Cas12a and CRISPR-Cas13a, seem to exhibit the required characteristics, in terms of high detection sensitivity and specificity, as well as simple and fast operation, to be considered as forceful detection tools for cancer diagnostics.^146^ It has been considered that CRISPR-Cas-based biosensing systems generate a new era for precise diagnosis of early-stage cancers. A new nanoparticle DNA-encoded nanosensor utilizing CRISPR-Cas-amplified urinary biomarkers has been designed and could enable early diagnosis of cancer with a simple urine test.^147,148^ The sensors, which can detect many different cancerous proteins, could also be used to distinguish the type of a tumor, whether a tumor has metastasized, or how it is responding to treatment.^147^

**Figure 14:**
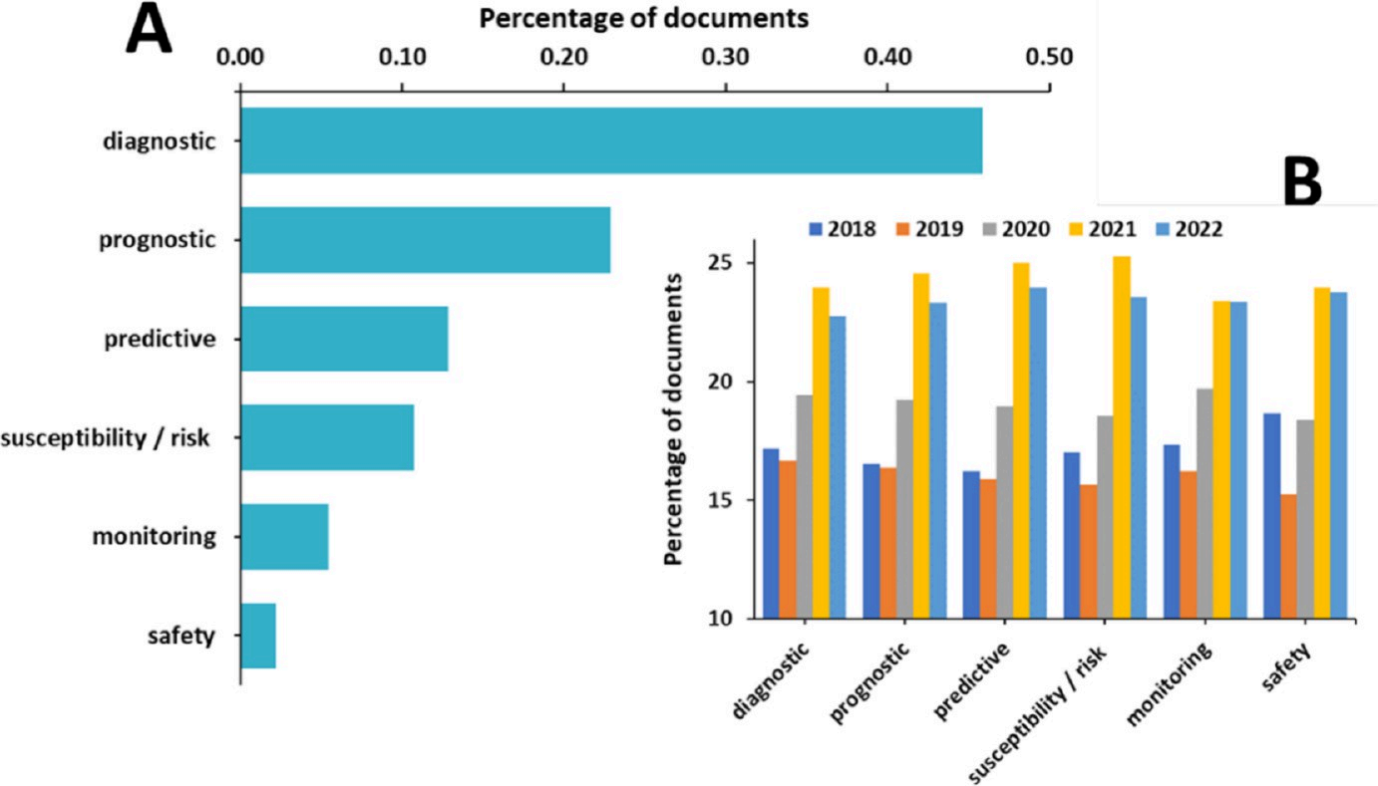
(A) Distribution of biomarker-related publications with respect to the biomarker functionality. (B) Yearly growth for the past 5 years (2018–2022).

Diagnostic biomarkers are the most widely used in cancers (Figure 14A). All biomarker types mark substantial growth in the past 2–3 years, but the susceptibility/risk biomarkers and the predictive biomarkers exhibit the highest yearly growth rate (Figure 14B).

**Figure 15:**
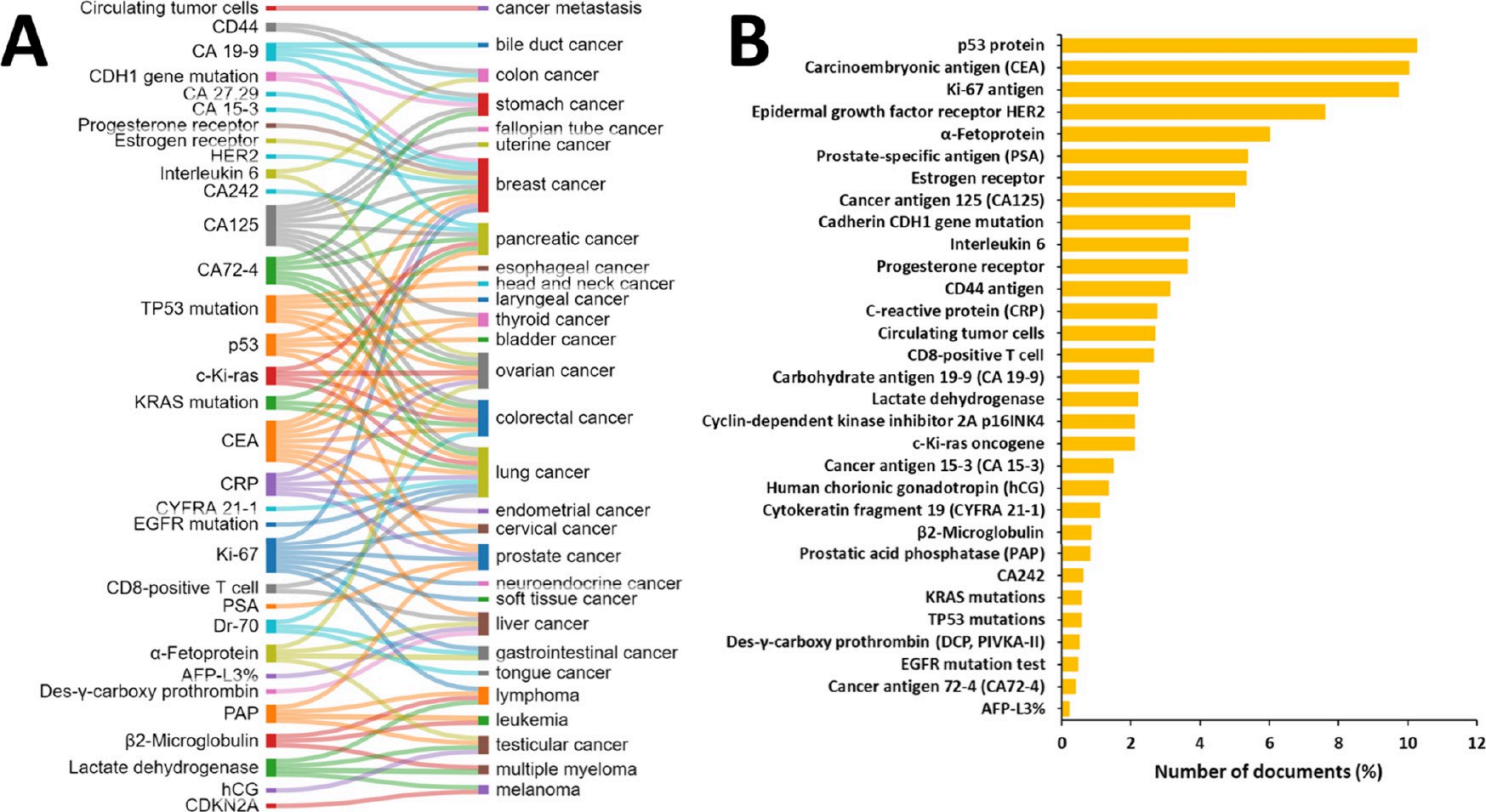
(A) Representative common cancer biomarkers and the corresponding types of cancer to which they are applied. (B) Number of documents (%) of the representative tumor markers in the CAS Content Collection.

Some biomarkers are specific for a particular type of cancer (e.g., CA 27.29, CA 15-3, and HER 2, which are used for breast cancer), while others are applied for a wider variety of malignancies (e.g., CA 125, Ki-67, and CEA). Figure 15 presents the relationship between a selection of common cancer biomarkers and the types of cancers they are applied for (Figure 15A), as well as the frequency of application of these biomarkers as reflected by the number of related documents in the CAS Content Collection (Figure 15B).

The p53 protein, the CEA, and the Ki-67 antigen have been explored in the highest number of documents (Figure 15B).

**p53** is one of the most frequently mutated genes in cancer, in the early phases of lung, skin, esophageal, and other cancers.^149−151^ p53 aggregates have been identified as prognostic marker in ovarian cancer.^152^ Mutant p53 is viewed as a biomarker for breast cancer^153^ as well as head and neck squamous cell carcinoma,^154^ and may help improve cancer surgery results.^155^

**Carcinoembryonic antigen (CEA)** is a serum marker commonly used for monitoring colorectal cancer, for evaluating prognosis, postoperative surveillance, and disease advance. It is also examined in other malignancies including medullary thyroid carcinoma, breast, liver, ovarian, pancreatic, and prostate cancers.^156^

**Ki-67** expression is strongly related to tumor cell proliferation and is commonly used as a proliferation prognostic and predictive indicator in a number of tumor types.^157^ Ki-67 appeared to be closely correlated with pancreatic tumor severity.^158^ There is a correlation between Ki-67 expression and patient survival in a number of other cancers, e.g., cervical and uterine cancers, non-Hodgkin’s lymphoma, and gastrointestinal cancer.^157^

**Alpha-fetoprotein (AFP)** is a glycoprotein, the expression of which is closely related to hepatocarcinogenesis, and it is a common tumor marker in a blood serum test upon screening for hepatocellular carcinoma (HCC).^159,160^ It is also applied in detecting other malignancies including hepatoblastoma, tumors of the ovary and testis, and gastrointestinal tract.^161^

**Carbohydrate antigen 19-9 (CA19-9)** is a regularly used biomarker for pancreatic ductal adenocarcinoma, a highly aggressive malignant cancer accounting for over 80% of pancreatic cancer occurrences.^162^ It is also elevated in other malignancies that include colon cancer, gastrointestinal malignancies, and cholangiocarcinoma (bile duct cancer).^161^

**Carbohydrate antigen 72-4 (CA72-4)** is a tumor-associated polymorphic epithelial mucin, which is highly expressed in adenocarcinomas, such as stomach, colon, breast, and lung adenocarcinomas, while it is rather low in normal tissues. It is applied as a conventional serum tumor marker for the diagnosis, monitoring, and prognosing of gastric cancer.^163^

**Human epidermal growth factor receptor-2 (HER2)** is a prognostic and predictive marker for breast cancer and is associated with poor clinical outcome. HER2 overexpression supposedly correlates with resistance to hormonal therapy and to CMF (cyclophosphamide, methotrexate, and fluorouracil) chemotherapy regimen.^164−166^

**Prostate-specific antigen (PSA)** is a protein produced by normal prostate cells. Although PSA is not a rightful diagnostic test for prostate cancer, rapidly escalating values of PSA in blood may be related to prostate cancer. Since tests for PSA levels in serum have been introduced into the clinic, early diagnosis of prostate cancer has been modernized, and much has been discovered about this assay. PSA tests helps in evaluating the response to therapy, monitoring tumor progression, as well as identifying men for whom a prostate biopsy would be appropriate.^167^ Data from the NIH indicates a 44% drop in prostate tumors mortality since PSA testing has become widely available in the early 1990s.^168,169^

**Estrogen/progesterone receptors** are breast cancer biomarkers, which are prognostic of outcomes, as well as predictive of response to certain therapies.^170^ These markers can be distinguished by using immunohistochemistry and fluorescence assays—fast and cost-effective detection methods. These molecular markers give the adequate prediction of the prognosis of cancer recurrence and progress.^171^

**Cancer antigen 125 (CA125)** is a commonly expressed by epithelial ovarian cancers, but also by various other gynecologic, such as cervical, endometrial, and fallopian tube cancers, as well as by some non-gynecologic cancers, such as pancreatic, breast, colon, lung, and thyroid cancers.^172−174^

**Figure 16:**
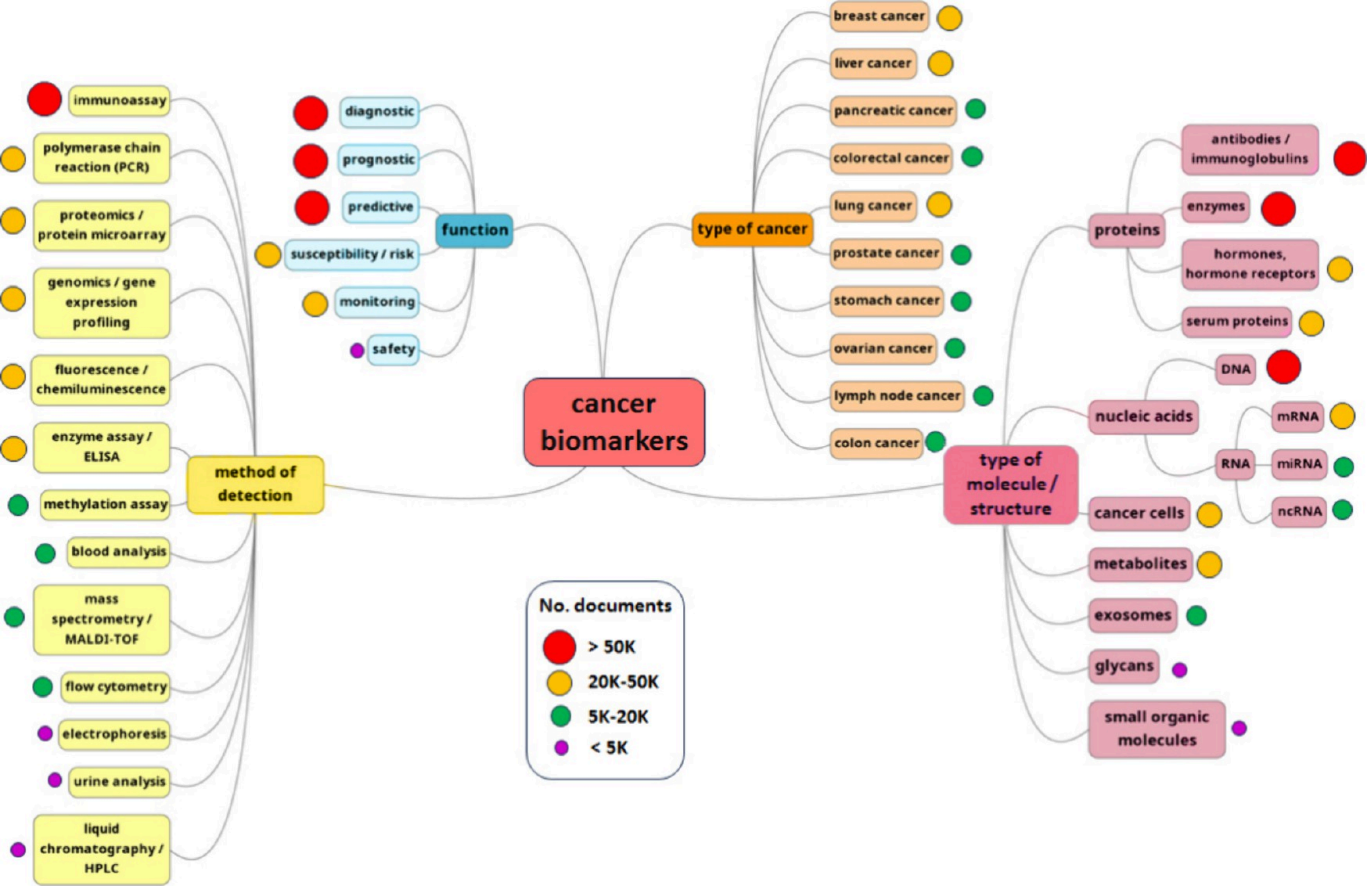
Mind map of the cancer biomarker research area with indication of the number of documents in each subcategory.

In Figure 16 we present a mind map of the cancer biomarkers research area, with indication of the number of documents related to each subcategory. The type of molecule/structure applied as a biomarker and its functionality are the areas attracting most attention.

### 2 Pancreatic Cancer Biomarkers

Pancreatic cancer is a relatively rare cancer with a global age-standardized incidence rate of 4.9. It is the 12th highest in global incidence with a 5-year prevalence of about 380,000 (WHO, Globocan 2020^175^).^176^ The low incidence of the cancer may have led to relatively little effort in resolving outstanding medical needs for the indication. However, there has been a steady increase in the incidence of pancreatic cancer globally, primarily observed in developed nations like North America and among younger individuals.^177,178^ It is estimated that by 2040, the number of new incidences would have increased by 70%. Several aspects are thought to contribute to this increase, including lifestyle factors like smoking, alcohol, obesity, and poor diet habits.^179,180^

**Figure 17:**
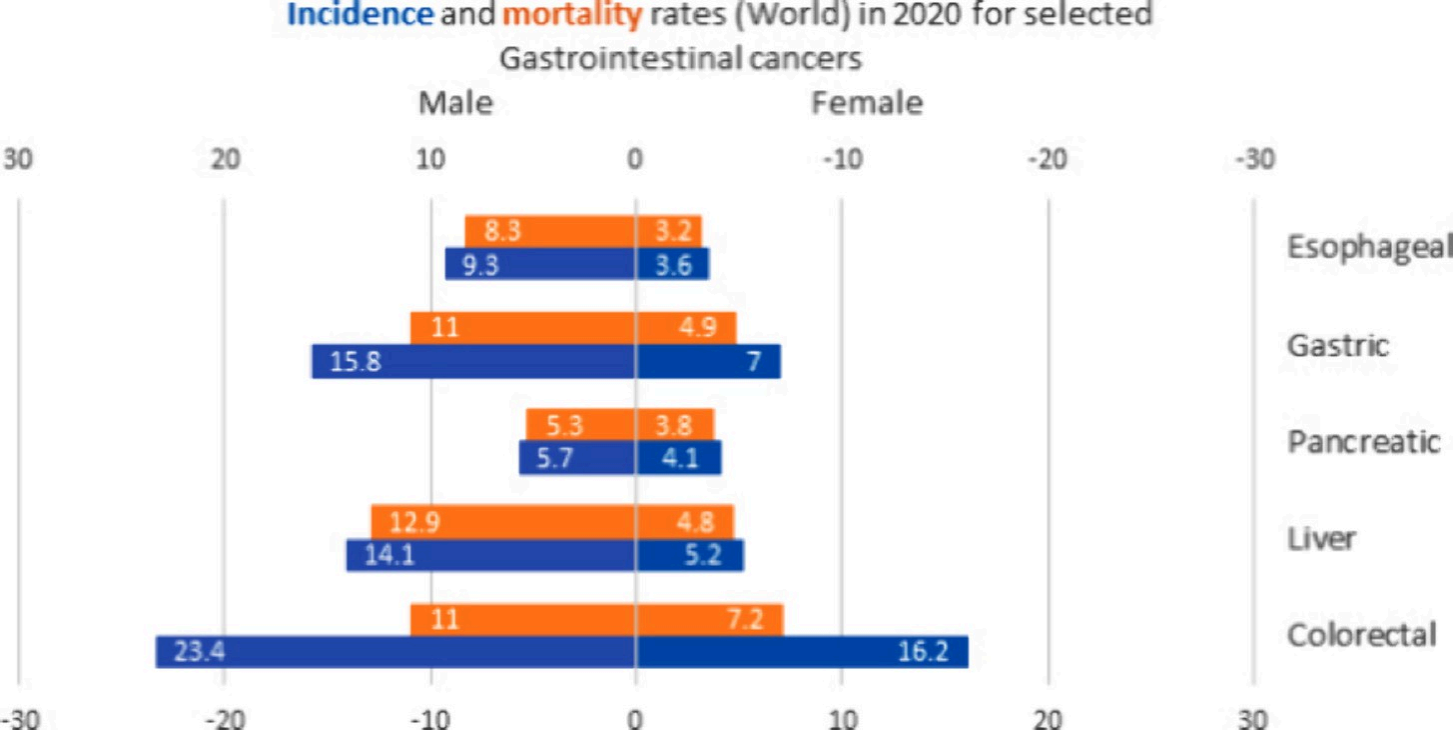
Global age-standardized incidence and mortality rates per 100,000 in selected gastrointestinal cancers, in both sexes. (Adapted from Globocan 2020.^85^)

Along with the consistent increase in incidence, pancreatic cancer also suffers from a very high mortality rate (Figure 17). At 4.7 it has the ninth highest global mortality rate and with a mortality rate of 7.6 accounts for the third highest deaths due to cancers in North America, after lung and colorectal cancer (WHO, Globocan 2020^175^)^176^ A similar situation exists in Europe with a mortality rate of 6.8. Pancreatic cancer has an extremely low survival rate estimated at 2–9% (5 years), associating it with a poor prognostic readout for patients. Compared to other gastrointestinal cancers, pancreatic and liver cancers are associated with some of the poorest survival prognosis.

The poor prognosis and high mortality associated with pancreatic cancer is largely attributed to late detection of the disease. Most cases present themselves at an advanced or metastatic stage, making it difficult to resect the tumor or control its spread. Early detection and diagnosis would mitigate this and help in improving survival. However, due to the low incidence and prevalence of this cancer, screening is recommended only for individuals at high risk due to familial cases of pancreatic cancer. Current early detection includes imaging modalities like EUS and MRI/MRCP (National Comprehensive Cancer Network, 2023).^181^

Some of the accepted biomarkers and risk factors for pancreatic cancer are listed in Table 1. Biomarker needs for pancreatic cancer can be divided into three aspects:

1. Biomarkers that will help identify high risk individuals so that they could be guided to regular screenings.
2. Improved screening biomarkers.
3. Biomarkers that will be able to differentiate better in benign vs neoplastic lesions.

**Table 1:** Representative Established Biomarkers and Risk Factors for Pancreatic Cancer

**Biomarker**	**Utility**	**Shortcomings**
Serum cancer antigen 19-9 (CA 19-9)^182^	Diagnostic, treatment response, monitoring	Low sensitivity and specificity
	• U.S. FDA cleared for use in routine monitoring of pancreatic cancer	

Imagining modalities: computed tomography (CT), magnetic resonance imaging/cholangiopancreatography (MRI/MRCP), endoscopic ultrasound (EUS), endoscopic ultrasound-guided fine-needle aspiration (EUS-FNA)^181^	EUS-FNA is the gold standard for pancreatic cancer diagnosis	Radiation exposure; low sensitivity for identifying solid pancreatic lesions less than 2 cm; may not detect metastasis

Inherited genetic syndromes, mutations: BRCA2, BRCA1, STK11, PRSS1, CDKN2A, ATM, MMR^183,184^	Disease risk	Useful in recognizing high-risk population

Blood glucose new-onset diabetes mellitus^185^	Disease risk	Useful in recognizing high-risk population
	• Patients with type 2 diabetes have a doubled risk of developing pancreatic cancer.	

Chronic pancreatitis^186^	Disease risk	Useful in recognizing high-risk population
	• Standardized incidence ratio of 22.2 (95% CI = 16.2–29.6) for the development of pancreatic cancer within 4 years, and 7.6 (95% CI = 6.0–9.7) within 24 years.	

KRAS, p16/CDKN2A, TP53, and SMAD4 somatic mutations^187^	Diagnostic	Measured often in ctDNA; cystic fluid often used as sample for next generation sequencing; has to be combined with other markers to improve specificity and sensitivity
	• Somatic mutations in KRAS and other genes help differentiate neoplastic growth.	

In the past decade, several studies have explored the potential for novel biomarkers for early detection of pancreatic cancer.^179,187,188^ These markers extend from metabolites to genetic mutations, standalone or combination panels and use varied sample sources with a general aim to move toward non-invasive testing.^189^ However, these markers lack stringent validation and are not included in treatment guidelines.^190^

To answer the need for diagnostic and risk markers in pancreatic cancer, it is important to sift through all the emerging markers and identify the ones which hold promise over others. An exploration of these biomarkers in Excelra’s database Biomarker Insights reveals all the emerging biomarkers and allows their selection and prioritization based on several factors, so that the ones with potential for clinical validation stand out.

### 3 Liver Cancer Biomarkers

Liver cancer or hepatocellular carcinoma (HCC) has the fourth highest mortality rate and represents an important public health concern, currently being the fourth cancer-related cause of death worldwide.^176^ The global age-standardized incidence rate of liver cancer is 9.5, much higher than pancreatic cancer. It is the sixth highest in global incidence with a 5-year prevalence of about 1 million (WHO, Globocan 2020^175^).^176^ The incidence is also higher in males than in females. Liver cancer has seen a steady increase in incidence in developed nations like North America. Like pancreatic cancer, liver cancer also suffers from a very high mortality rate. At 8.7, it has the fourth highest global mortality rate closely following colorectal cancer (WHO, Globocan 2020^175^).^176^

Just as with pancreatic cancer, the identification of useful biomarkers for surveillance and early diagnosis of HCC, is lacking. Some risk factors for HCC are well established such as chronic hepatitis B (HBV) or C virus (HCV) infection and any chronic liver disease with severe fibrosis or cirrhosis. Clinical practice guidelines include screening techniques through imaging. However, no molecular markers have been found to be highly specific or sensitive.^191,192^ While several biomarkers have been explored, further validation is lacking for most. Table 2 lists some of the established and most common biomarkers used in liver cancer.

**Table 2:** Representative Established Biomarkers and Risk Factors for Liver Cancer

**Biomarker**	**Utility**	**Shortcomings**
Ultrasonography, CT, MRI^191−193^	Screening at 6-month intervals for individuals at high risk	Operator driven and likely to have lab variations

AFP^191−193^	HCC surveillance	Low specificity; upregulated in chronic hepatitis, intrahepatic cholangiocarcinoma, and embryonic tumors
	• AFP had the highest AUROC for early HCC diagnosis.	

AFP-L3 (with or without combining with AFP)^194^	Combination of AFP (highly sensitive assay; cutoff >5 ng/mL) and AFP-L3 (cutoff >4%) showed the highest AUROC value (0.83) when compared to any single biomarker	Needs more clinical validation; 95% and 71% of patients had positive values of %AFP-L3 at 3 and 6 months before diagnosis, respectively; sub-optimal in terms of cost-effectiveness for routine surveillance of early HCC

Des-γ-carboxy prothrombin (DCP)^193,195,196^	A combination of AFP and DCP may give improved results	Needs more clinical validation

HBV, HCV infection, liver cirrhosis, fibrosis^191^	Risk assessment; marked for regular surveillance	

### 4 Biomarker Insights for Pancreatic and Liver Cancer from the Excelra Biomarker Insights Dataset

Excelra’s Biomarker Insights dataset^106^ contains manually curated information around biomarkers for selected disease indications. Information is captured for about 85 distinct fields allowing one to explore connections between disease, biomarker, drug, and clinical outcomes. Biomarkers are largely curated from research articles, clinical trials, guidelines, and drug labels. They are presented as an interactive dashboard and networks, allowing easy exploration and emergence of new insights.

**Figure 18:**
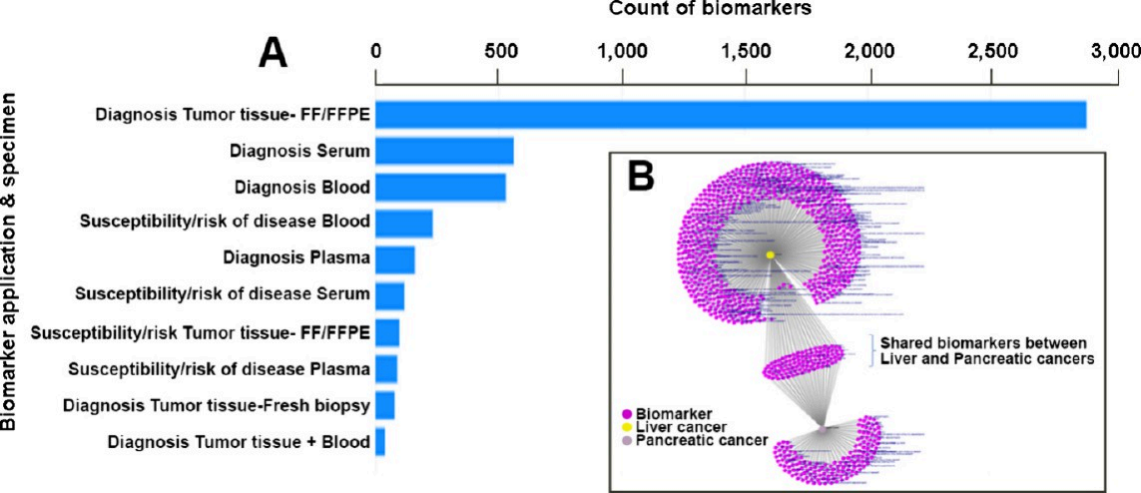
(A) Top 10 biomarker application-specimen combinations among the diagnostic and risk biomarkers of pancreatic and liver cancer (FF, fresh frozen; FFPE, formalin-fixed, paraffin-embedded). (B) Diagnostic and risk biomarkers exclusive and shared between liver (yellow node) and pancreatic (light pink node).

An extraction of diagnostic and risk markers from this dataset yields 4,160 biomarkers for pancreatic and liver cancers. Table 3 gives statistics on some core entities and tags related to biomarkers from the dataset. When assessing potential markers that can be used for diagnosis or risk classification, there are a total of 1,163 and 3,582 in pancreatic and liver cancer, respectively. These markers have been measured using varied specimens. Figure 18A shows the representation of the top 10 categories of diagnostic or risk biomarkers in combination to the specimen they were measured in. Figure 18B is a graphical representation on how the biomarkers are shared between the two indications. There are 1,007 biomarkers associated exclusively with pancreatic cancer and 3,094 exclusive to liver cancer. 425 markers are shared between the two indications. It would be interesting and valuable to assess promising markers from that shared set which could be utilized in both indications.

**Table 3:** Statistics on Certain Core Entities and Tags Related to Biomarkers from the Excelra Biomarker Insights Dataset^106^

	**Biomarker Count**	
**Specification**	**Pancreatic cancer**	**Liver cancer**
Total	1927	5752
Diagnostic application	1050	3218
Disease risk application	113	364
Non-invasive/minimally invasive sampling	366 (189 proteins)	1107 (279 proteins)

**Figure 19:**
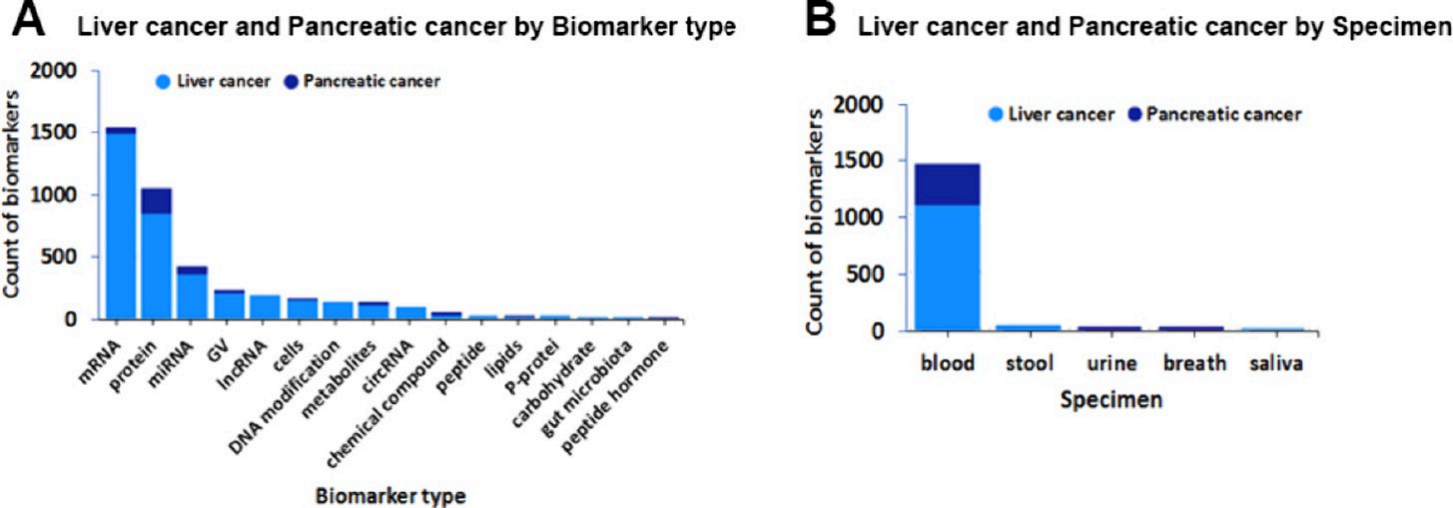
Counts of biomarkers for the pancreatic and liver cancers sorted by biomarker type (A) and specimen type (B). GV = genetic variants.

Figure 19 shows the count of biomarkers classified by biomarker type (Figure 19A) and by specimen (Figure 19B). mRNA markers are the leading type markers for liver cancer, while proteins are the most abundant type markers for pancreatic cancer. Blood samples dominate for both type of cancers.

The large number of relevant markers present in the Biomarker Insights dataset^106^ are captured from research articles spanning the past 7 years. To assess promising markers within these, a scoring of the diagnostic and risk markers is required in order to prioritize the ones which can be considered for further validation and diagnostic application. A validation score is calculated for each of these markers based on five contributing factors.^197^ These factors are weighted according to their relative value in determining biomarker–disease association:

1. Biomarker qualification status: Highest weight is given to pre-existing regulatory qualification of the biomarker, its presence as a companion diagnostic, and presence of the biomarker in any drug-label or clinical guidelines.
2. Number of supporting articles: This is a count of number of distinct articles that provide evidence to the association of the biomarker to the disease.
3. Study category of those articles: This component rates the article category e.g., registered clinical trials get a higher rating than a case study.
4. Combined number of patients/samples referenced in the study: This component scores the number of patients recruited in a study, e.g., a study with 500 samples would rank higher than a study with 50.
5. Multiple contexts of use for the biomarker: Multiple applications tagged with the biomarker in the disease will positively impact its scoring. For example, a biomarker with both predictive and prognostic applications will rank higher than a biomarker with only prognostic application.

In addition, the markers are also scored for the clinical outcome they measure, in this case diagnosis and risk. This scoring is a summation of the number of studies that concluded a positive or negative association of the marker with the outcome. A positively associated marker is high (expression level, or presence of variant) when the disease is present and the negatively associated one low.

**Figure 20:**
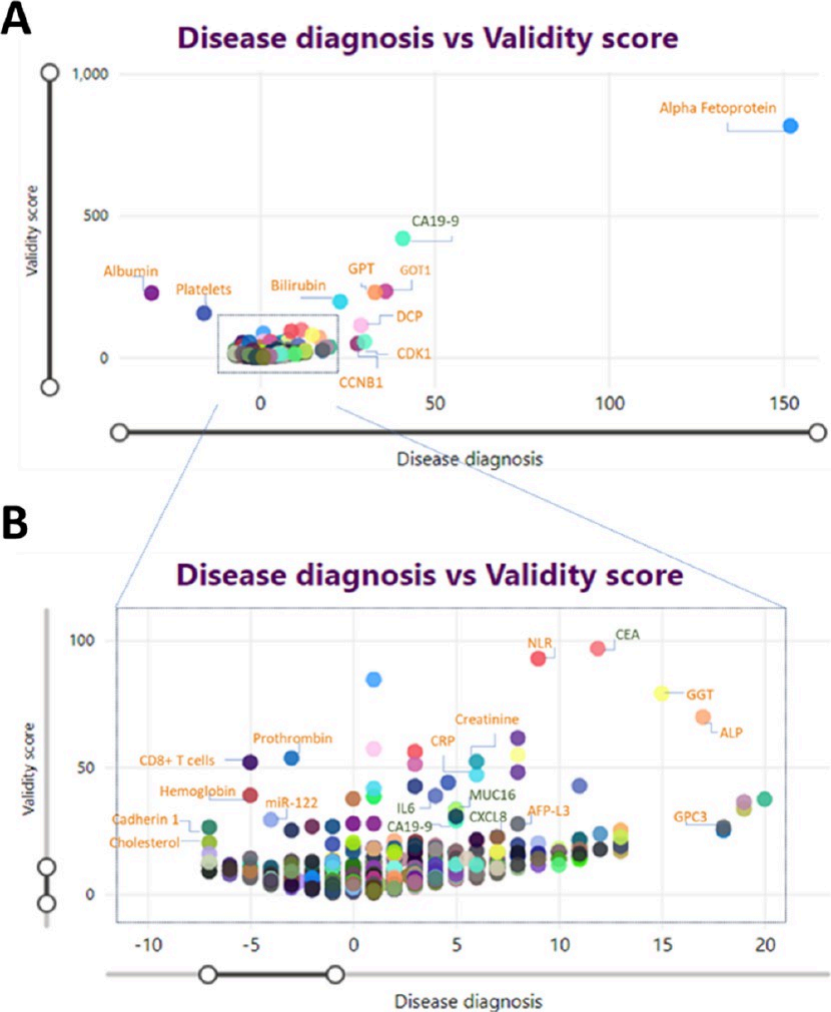
Diagnostic and risk biomarkers of pancreatic (green) and liver (orange) cancers: biomarker landscape scores for validity (*Y*-axis) and disease diagnosis association (*X*-axis). Panel A is the complete plot, and panel B represents a zoomed-in view.

On this basis the selected markers are landscaped for prioritization and selection as shown in Figure 20A, with a zoomed-in view in Figure 20B. Some key biomarkers are highlighted which are already on the market and being used as established markers while several others which could be promising markers are highlighted in Figure 20B. Table S1 in the Supporting Information shows a list of all these markers along with the information on the cancer they are associated with, and the kind of sample used to measure them.

**Figure 21:**
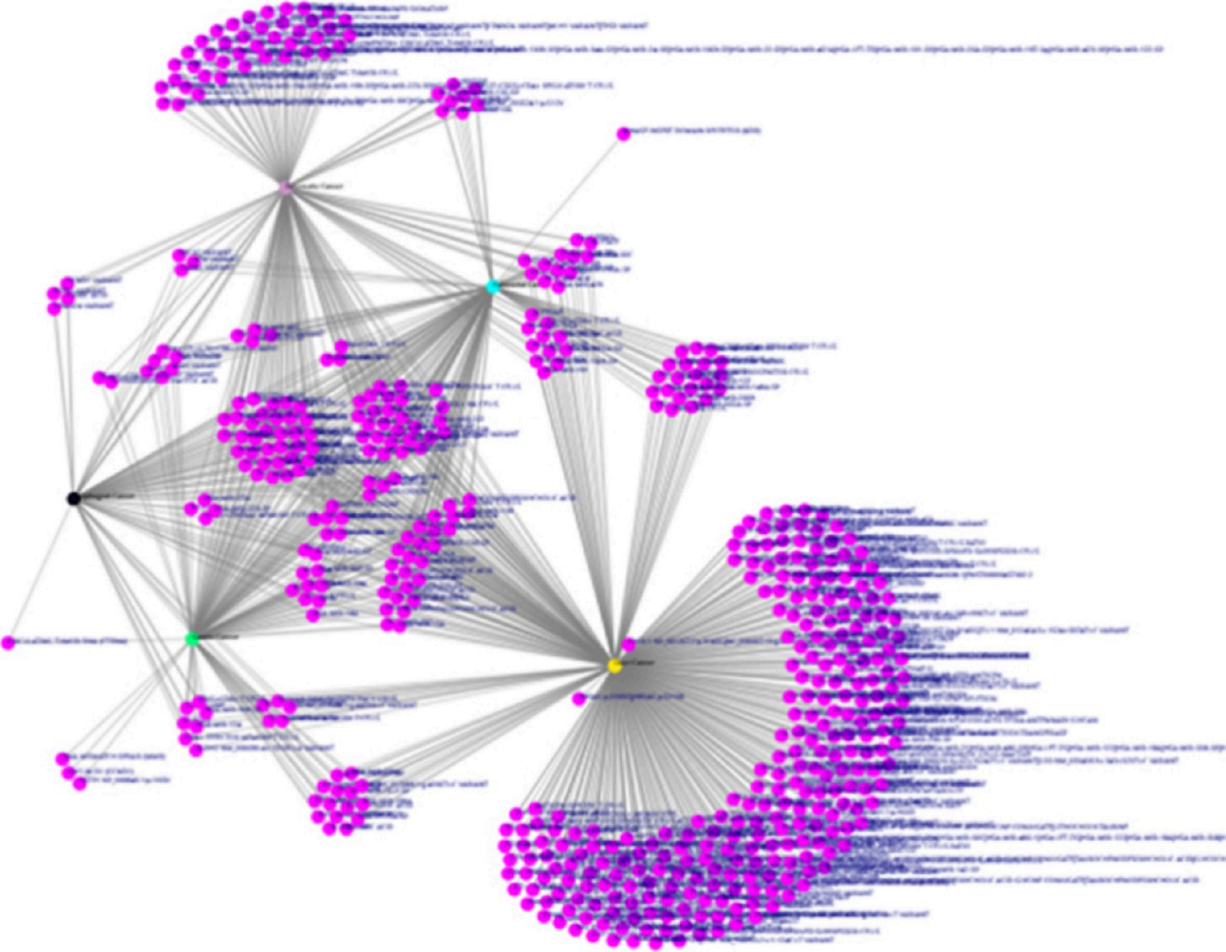
Interaction network of diagnostic and risk biomarkers that can be measured through non-invasive sampling. The biomarkers (magenta nodes) and their links with five gastrointestinal cancers are depicted. For an interactive view of this network visit ref (198).

From within this list, markers which are measured in specimens collected using non-invasive or minimally invasive techniques were further shortlisted. A total of 1992 unique biomarkers were used to build a network graph to put them in context with a total of five gastrointestinal cancers (Figure 21). Such an analysis helps understand the specificity of these biomarkers for the disease of choice as well as the potential of a biomarker to work across several indications. The network can be viewed in an interactive way at ref (198).

### 5 Capital Investment

**Figure 22:**
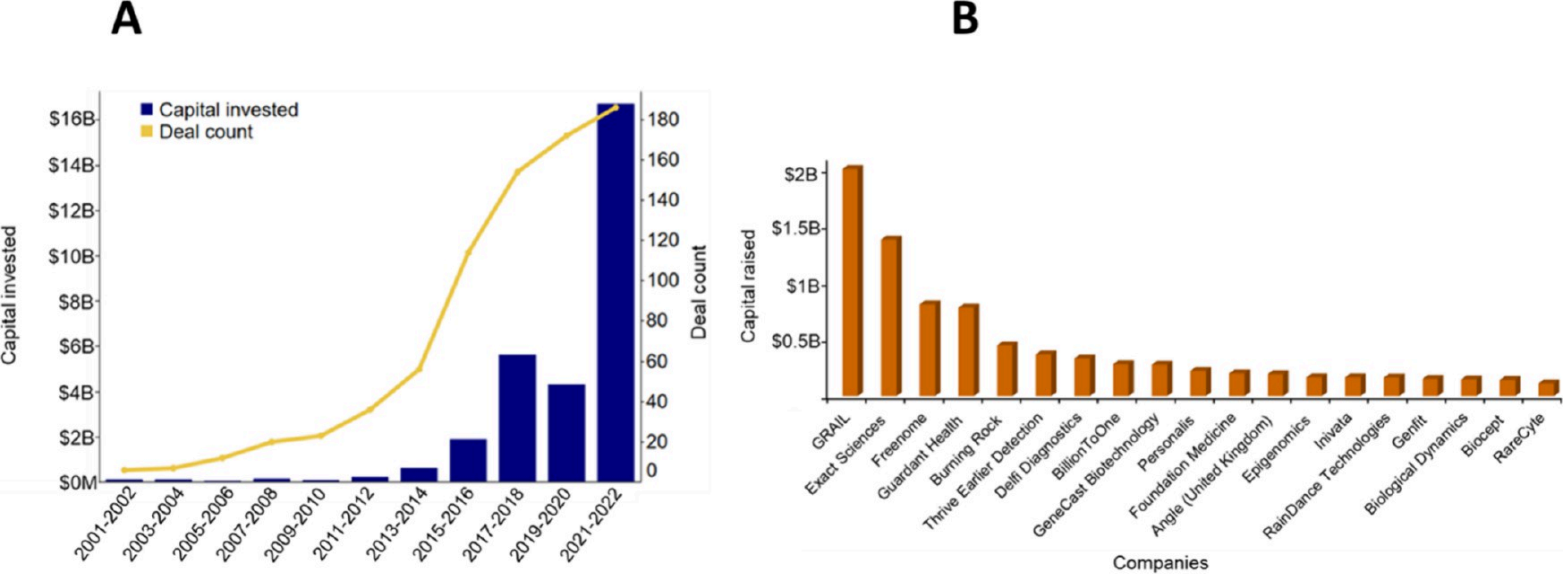
Commercial interest in early cancer diagnostics (data sourced from PitchBook). (A) Capital invested and deals related to early cancer diagnostics for the past 2 decades (2000–2022). (B) Leading companies in terms of capital raised in the field of early cancer diagnostics from 2000 to 2022.

Capital investment data from Pitchbook^199^—an online platform for investment data, reveals a steady increase in invested capital and financial deals over the past 20 years for early cancer diagnostics (Figure 22A). Interestingly, an exception appears to be the capital investment profile from 2019 to 2020, which shows a slight dip in the amount of money invested indicating the reduction in investment during this period (Figure 22A), the exact reason for which remains unknown. Companies like GRAIL that work on multi-cancer early detection (MCED) testing has raised the highest capital (∼$2B) in the past 20 years (Figure 22B). GRAIL has developed the first clinically validated MCED test—the Galleri test that can help in early cancer detection of over 50 types of cancers from a single blood draw.^200^ Other companies such as Exact Sciences^201^ and Freenome,^202^ which work on early cancer detection, have raised a considerable capital investment in the past 2 decades.

**Figure 23:**
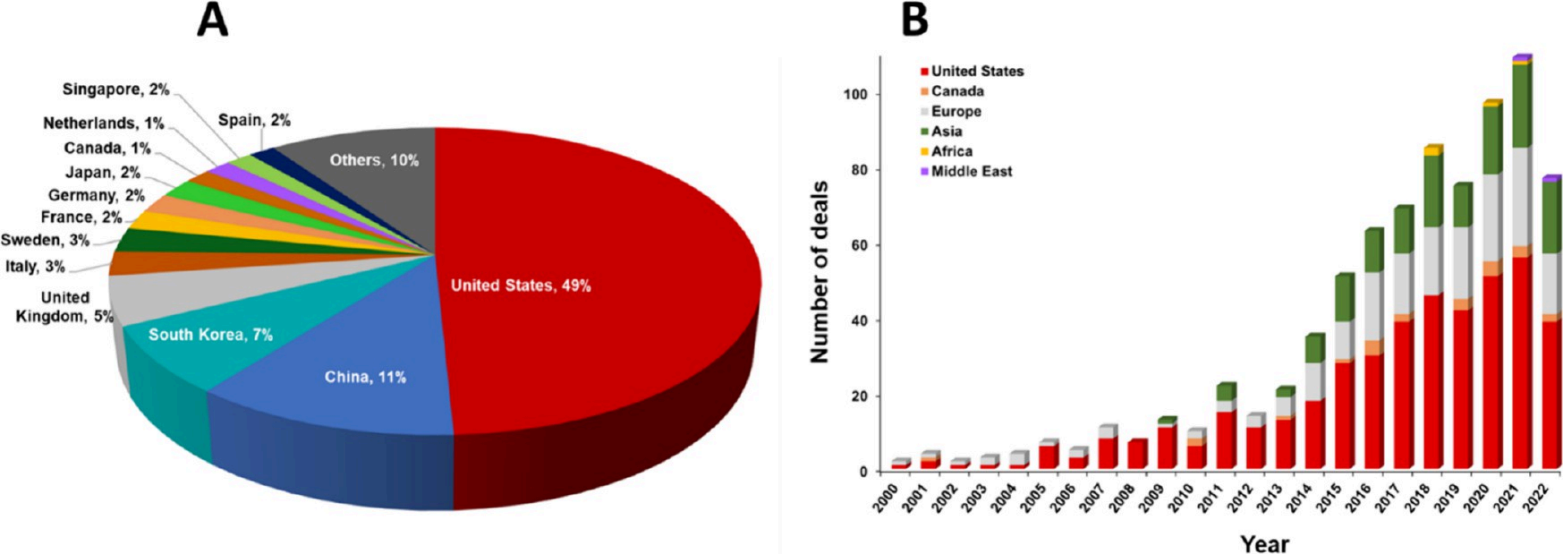
(A) Geographical distribution of the number of companies engaged in the field of early cancer diagnostics. (B) Growth in the number of deals in the field of early cancer diagnostics by different countries/regions or regions over a period from 2000 to 2022.

In terms of geographical distribution of capital investment in the field of early cancer detection, the U.S. leads with respect to capital investment from 2000 to 2023, followed by China and South Korea (Figure 23A). The investment in the U.S. is ∼5 times that of China and ∼7 times that of South Korea. Growth in the number of deals made over the past 2 decades for the few leading countries/regions shows a steady increase, excluding a minor dip seen in the number of deals in 2022 (Figure 23B). This trend indicates a continued interest of companies in the field of early cancer detection. Unsurprisingly, the U.S. has the highest number of deals followed by Europe and Asia (Figure 23B). Regions/countries/regions from the Middle East and Africa also show a presence in the past 5 years (2018 onward).

### 6 Commercial Development of Early Cancer Screening Assays

Companies worldwide are researching and creating tests to detect pancreatic and liver cancers at early stages to save lives and improve patient outcomes. In terms of regulatory approval, the U.S. FDA categorizes medical devices under different classes based on risk. Class I (low risk) will require no FDA approval, Class II (moderate risk) will require FDA 510(k) clearance, and Class III (high risk) will require FDA approval. There is also the FDA breakthrough device program that speeds up development, assessment, and review for pre-market approval, 510(k) clearance, and De Novo marketing authorization for devices that provide more effective diagnosis of cancer than other devices currently on the market.^203^

A few tests that have received such FDA designations are Thermo Fisher’s BRAHMS CgA II KRYPTOR test, that is the first and only FDA-cleared, fully automated chromogranin A assay used to detect gastroenteropancreatic neuroendocrine tumors^204^ and also an AFP-L3% immunological test system that was cleared by the FDA in 2005 for the risk assessment of HCC.^205^ A few other tests have also received FDA breakthrough device designation such as GRAIL’s Galleri test in 2019^206^ and more recently in 2023, Burning Rock Dx’s OverC test.^207^ A selection of companies that are researching early cancer detection for liver and/or pancreatic cancer are explored in Table 4 to show the development and diversity within this emerging field.

**Table 4:** Selection of Organizations Developing Early Screening Tests for Pancreatic and Liver Cancers

**Organization, Location**	**Summary**
20/20 GeneSystems, USA	Developer of OneTest which is a blood test and ML algorithm combined to aid in the detection of multiple cancers including pancreatic, colon, lung, kidney, ovarian, and bladder cancer.^208^
Acuresis Bio, South Korea	Provider of a pancreatic cancer diagnostic kit. This company is currently operating in stealth mode.^209^
Beken Bio, USA	Developer of liquid biopsy-based cancer detection utilizing cancer-specific antigens transported by extracellular vesicles and a ML/AI-based algorithm. Their laboratory-developed test called 3DReveal is under clinical validation for the early detection of ovarian cancer, with expansion into lung, pancreatic, and colorectal cancers within the next 18 months.^210^ (C. M., personal communication, Oct 17, 2023)
B.R.A.H.M.S., Germany	Part of Thermo Fisher Scientific. Their CgA II KRYPTOR test is used for the early identification of gastroenteropancreatic neuroendocrine tumors. It is the first FDA-cleared, automated immunofluorescent assay for the quantitative determination of the concentration of chromogranin A in human serum.^204^
Burning Rock Dx, USA	Developer of the OverC Multi-Cancer Detection Blood Test that tests for five cancer types, including liver and pancreatic. The cfDNA methylation MCED technology is aided by ML and received FDA breakthrough device designation in January 2023.^211^
Chip Diagnostics, USA	Developer of exosome-based diagnostics making early cancer diagnosis possible. Their pancreatic cancer test utilizes biomarkers, exosome-based miRNA, cfDNA, and CA19-9 proteins and is in clinical development.^212^ Their liver cancer test is currently under preclinical development.^213^
ClearNote Health, USA	The company’s current focus is on pancreatic cancer with their Avantect pancreatic cancer test. The test utilizes an epigenomics approach and measures levels of the biomarker 5-hydroxymethylcytosine to detect pancreatic cancer. They are also developing a targeted multi-cancer test.^214^
Delfi Diagnostics, USA	Utilizes a genome-wide fragmentomic-based approach along with advanced ML for early cancer detection.^215^
Detectiome, United Arab Emirates	Developer of an AI-enabled liquid biopsy solution designed to detect multiple cancers in the early stages.^216^
Enrich Bioscience, Canada	The company’s technology utilizes DNA methylation to detect different types of cancer, including lung, liver, bladder, prostate, colorectal, breast, pancreatic, thyroid, gastric cancer and leukemia, from extracted DNA using a non-invasive blood test.^217^
Exact Sciences, USA	Developer of Oncoguard Liver, a genetic-based treatment selection test. They are also building a multi-cancer early screening test, Cancerguard, to detect over 14 cancers.^218^
Glycotest, USA	Developer of a non-invasive blood test to provide information on the likelihood of HCC. It measures the amount of monosaccharide fucose that appears abnormally high on certain glycoproteins to indicate the likelihood of disease.^219^
GRAIL, USA	Developer of a MCED test kit, Galleri, designed to detect more than 50 types of cancer through non-invasive blood analysis. Galleri received FDA breakthrough device designation in 2019.^206^
Helio Genomics, USA	Developer of HelioLiver, a multi-analyte blood test that evaluates cfDNA methylation patterns, serum protein markers, and demographic information for the detection of HCC.^220^
HKG Epitherapeutics, Hong Kong	Developer of EpiLiver for the early detection of liver cancer utilizing the detection of DNA methylation changes in ctDNA. They use the science of epigenetics for the early detection of cancer.^221^
Immunovia, Sweden	A diagnostics company that recently ceased commercial production of their early pancreatic cancer test IMMray PanCan-d. They are now focusing their resources on the further development and clinical testing of the company’s promising next-generation pancreatic cancer detection test which will reduce reliance on the biomarker CA19-9.^222^
MiRXES, Singapore	Developer of an ID3EAL reverse transcription quantitative PCR platform for detection of miRNAs and other ncRNAs. This technology is the foundation on which their MCED clinical pipeline is built, which includes liver and pancreatic cancer detection.^223^
Owlstone Medical, UK	Developer of a disease breathalyzer test for liver disease and HCC among other diseases.^224^
PinPoint, UK	The company’s platform uses ML to combine information from multiple cancer biomarkers and patient information to help identify patients who have a high probability of cancer diagnosis.^225^
SeekIn Medical, China	The company’s early cancer detection test utilizes a ctDNA mutation-centered analytical approach that is combined with advanced ML and AI. SeekInCare is their test for early detection of pancreatic cancer. OncoSeek, their MCED test which includes both pancreatic and liver cancer, received European CE mark of approval in September 2022.^226^
Tzar Labs, Singapore	Developer of a non-invasive HCC detection test. The company’s technology helps in the detection of tumors by using embryonic-like stem cells and cancer stem cells for diagnostics, thereby helping to detect cancers, including pancreatic and liver, even years before symptoms occur.^227^
Universal Diagnostics, Spain	The company combines proprietary tools with ML algorithms to offer minimally invasive, blood-based tests that can detect the disease in its earliest stages and forms. Their Signal-G test screens for pancreatic, liver, and gastric cancers.^228^

### 7 Clinical Trials for Early Cancer Diagnosis of Pancreatic and Liver Cancers

**Figure 24:**
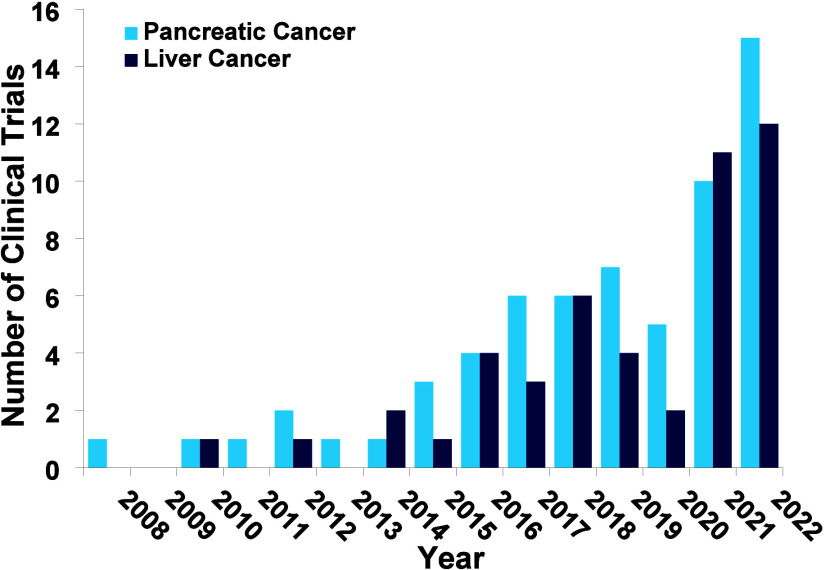
Number of early cancer diagnostic clinical trials focused on pancreatic and liver cancer by year.

A representative selection of early cancer diagnostic clinical trials focused on the diagnosis of pancreatic and liver cancers are examined within this section to gain an overall view of the past, present, and future state of clinical development. A selection of around 100 clinical trials^229^ are examined against time, disease indication, and trial status. Early cancer diagnostic testing for pancreatic and liver cancers are just starting to see increased numbers in clinical development, with Figure 24 showing a steady growth for pancreatic cancer starting in 2015 with a decrease in research in 2020 and then sharply increasing for the past few years. Liver cancer has seen oscillating growth with also a decrease in 2020 and then mirrors pancreatic cancer’s sharp increase in activity within the past few years.

**Figure 25:**
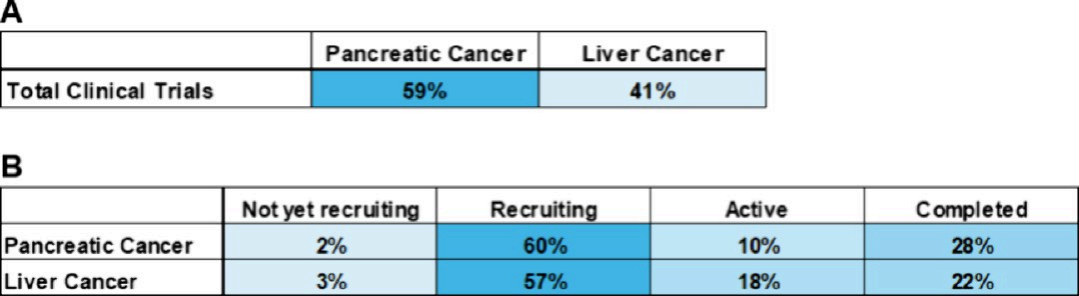
(A) Percentage of each disease indication for early cancer diagnostic clinical trials focused on pancreatic and liver cancer. (B) Percentage of early cancer diagnostic clinical trials focused on pancreatic and liver cancer in various clinical trial statuses.

Analysis of the above clinical trials by disease indication reveals that 59% of these trials are focused on the diagnosis of pancreatic cancer with 41% focused on the diagnosis of liver cancer (Figure 25A). Further analysis reveals the clinical trials statuses of early cancer diagnostic clinical trials focused on pancreatic and liver cancers. Most clinical trials for both indications are currently in recruiting status; gathering participants while getting ready to move into active status (Figure 25B). 60% of pancreatic cancer trials and 57% of liver cancer trials are in recruiting status. With less than 30% of trials being completed (Figure 25B), we can expect to see more active clinical trials soon. Currently only 10% of pancreatic cancer trials are in the active state with 18% active for liver cancer.

Finally, representative clinical trials examining early cancer diagnostics focused on pancreatic and liver cancer are highlighted in Table 5 and examined in further detail below to showcase the diversity and progress within the clinical development pipeline.

**Table 5:** Highlighted Early Cancer Diagnostic Clinical Trials Focused on Pancreatic and Liver Cancer

**Indication**	**Intervention**	**Status**	**Sponsor**	**NCT Number**
Multiple cancers, including pancreatic and liver	Galleri Multi-Cancer Early Detection Test	Recruiting	GRAIL	NCT05155605
Multiple cancers, including pancreatic and liver	MiRXES Multi-Cancer Screening Test	Recruiting	MiRXES	NCT05633342
Multiple cancers, including pancreatic and liver	Galleri Multi-Cancer Early Detection Test	Completed	GRAIL	NCT04241796
Pancreatic cancer	Pancreatic Cancer Early Detection Test	Recruiting	Ruijin Hospital/Burning Rock Dx	NCT05556603
Pancreatic cancer	Early Pancreatic Cancer Detection Tool	Active	Peking Union Medical College Hospital	NCT05689138
Gastroenteropancreatic neuroendocrine tumors	NETest	Completed	H. Lee Moffitt Cancer Center and Research Institute	NCT02948946
HCC	Glycotest HCC Panel	Recruiting	Glycotest	NCT03878550
HCC	HelioLiver	Active	Helio Genomics	NCT03694600
HCC	HelioLiver	Complete	Helio Genomics	NCT05059665
Liver disease, HCC	ReCIVA	Complete	Owlstone Medical	NCT03756597

GRAIL has developed a MCED blood test called Galleri that includes pancreatic and liver cancers. Their PATHFINDER clinical trial (NCT04241796) included 6662 participants and assessed the time required and the diagnostic testing necessary to confirm the presence or absence of cancer. Recently published results revealed that 1.4% of participants received a positive result. Of those positive results, 38% were true positives and would go on to get a positive cancer diagnosis with further screening.^230^ GRAIL will continue this research with a PATHFIDER 2 clinical trial (NCT05155605), currently recruiting, to further access the safety and performance of the Galleri test. Mirxes is another company researching MCED and is currently recruiting for their CADENCE (CAncer Detected Early caN be CureEd) clinical trial (NCT05633342). This study will develop a blood-based multi-cancer screening test to detect nine of the most prevalent cancers in Singapore including pancreatic and liver. The three-year study will investigate miRNA expression with other biomarkers such as DNA methylation among 20,000 participants.

Early cancer detection tests that focus solely on pancreatic cancer are also being investigated. Ruijin hospital is collaborating with Burning Rock Dx for an upcoming clinical trial that is recruiting over 7,000 participants. The ASCEND-PANCREATIC multi-omics, observational study (NCT05556603) will aim to detect pancreatic cancer with combined assays of biomarkers for cfDNA methylation, ctDNA mutations, serum protein markers, and miRNA. Peking Union Medical College Hospital is also conducting an active clinical trial (NCT05689138) analyzing the gut and fecal microbiome of participants with and without pancreatic cancer to establish an early detection tool and discover diagnostic biomarkers. The Moffitt Cancer Center also researched early pancreatic diagnostics with a completed clinical trial (NCT02948946) evaluating a blood based multi-gene RNA transcript assay for gastroenteropancreatic neuroendocrine tumors among 100 cancer and healthy participants. The NETest was found to be highly sensitive (98%) and specific with 48 true positive results and one false negative.^231^

Lastly, early cancer detection tests focused solely on liver cancer are examined. Glycotest seeks to validate their Glycotest HCC panel for the early detection of hepatocellular carcinoma. Their large multi-center clinical trial (NCT03878550) will include patients with early-stage HCC against non-cirrhosis and cirrhotic patients without HCC. The main objective will determine if the Glycotest HCC panel will be superior to current AFP surveillance techniques. Helio Genomics also researched their HelioLiver test in a completed clinical trial called the ENCORE study (NCT05059665). They found that HelioLiver showed superior performance to current AFP and GALAD (gender, age, AFP-L3, AFP, des-gamma-carboxy prothrombin) techniques.^232^ They are continuing their research with an active clinical trial (NCT03694600) to further explore HelioLiver’s performance in detection of DNA methylation profiles of cfDNA whole blood specimens. Finally, Owlstone medical has researched their breathalyzer for breath biopsy testing of liver disease and HCC in a recently completed clinical trial NCT03756597. Their breath biopsy system measures volatile organic compounds (VOCs) for disease surveillance. Endogenous VOCs are produced throughout the body and are distributed in the bloodstream, they exchange in the lungs and are exhaled. Exhaled VOCs contribute to biomarker discovery by affording a source of valuable biomarkers with distinct associations to the body metabolism.^233^ Results from this trial are encouraging, revealing 7 known and novel VOCs for a liver disease biomarker panel that can be utilized for disease detection.^234^

## Outlook, Challenges, and Perspectives

### Perspectives

The development of biomarkers for early cancer diagnosis holds significant promise in improving patient outcomes and transforming cancer care. Overall, the perspectives in developing biomarkers for early cancer diagnosis are focused on leveraging advancements in genomics, molecular profiling, non-invasive techniques, computational analysis, and collaborative research to enhance early detection, improve diagnostic accuracy, and enable personalized treatment approaches. Continued investment in research and technology development, along with regulatory support, will be key to realizing the full potential of biomarkers in cancer care.

- Advances in genomic sequencing technologies enable the identification of specific genetic alterations and mutations associated with different types of cancers. Integrating **genomic and molecular profiling** data can lead to the discovery of new biomarkers and personalized treatment approaches tailored to individual patients.^235,236^
- **Liquid biopsies** involve the analysis of circulating tumor cells (CTCs), cell-free DNA (cfDNA), exosomes, and other biomolecules and structures found in body fluids such as blood or urine. Liquid biopsies offer a non-invasive and real-time approach for detecting cancer-specific alterations, monitoring treatment response, and detecting minimal residual disease or cancer recurrence.^237,238^
- Integrating **multiple omics** data, including genomics, transcriptomics, proteomics, and metabolomics, can provide a more comprehensive understanding of cancer biology and help identify novel biomarkers. Analyzing multiple layers of molecular information can enhance diagnostic accuracy and predictive capabilities.^239−243^
- The application of **artificial intelligence (AI) and machine learning (ML)** algorithms can aid in the analysis and interpretation of complex biomarker data. AI can assist in identifying patterns, predicting disease outcomes, and improving the accuracy and efficiency of cancer diagnosis.^244−248^
- **MicroRNAs (miRNAs) and other non-coding RNAs (ncRNAs)** have shown promise as potential biomarkers due to their involvement in gene regulation and their dysregulation in various cancers. Further research into the functional roles of these molecules can lead to the discovery of novel biomarkers for early cancer detection.^29,249−251^
- Combining **imaging techniques** such as magnetic resonance imaging (MRI), positron emission tomography (PET), and computed tomography (CT) scans with molecular biomarkers can provide a comprehensive and multi-dimensional view of cancer. This integrated approach can improve diagnostic accuracy and guide treatment decisions.^36,37,252,253^
- Developing biomarkers that allow for **longitudinal monitoring and real-time assessment** of treatment response is crucial. Dynamic biomarkers that reflect the evolving state of the disease can provide valuable information for early detection, treatment optimization, and monitoring disease progression.^254,255^
- **Collaborative efforts and data sharing** among researchers, clinicians, and institutions are vital for biomarker development. Large-scale multi-center studies and data repositories facilitate the validation and reproducibility of biomarkers and foster innovation in the field.
- Biomarkers can be utilized for **personalized risk assessment** by combining individual risk factors such as genetic predisposition, lifestyle, and environmental exposures. This approach can enable tailored screening strategies and early intervention for individuals at high risk of developing specific cancers.^256−259^

### Challenges and Roadblocks

Developing biomarkers for early cancer diagnosis is a truly complex and challenging task. Certain roadblocks can impede their development and clinical application. Cancer is a highly heterogeneous disease, meaning that it can vary in its molecular and genetic characteristics even within the same type of cancer. Identifying biomarkers that accurately represent this heterogeneity and can be universally applicable is a significant challenge. Many biomarkers may be elevated in various conditions, including non-cancerous diseases. This lack of specificity can lead to false-positive results and unnecessary follow-up testing or procedures, causing anxiety and increasing healthcare costs. Also, biomarker levels can vary significantly between individuals due to factors such as age, sex, ethnicity, lifestyle, and co-morbidities. This variability can make it challenging to establish accurate cutoff values for diagnosis or risk assessment. Achieving both high sensitivity (detecting true positives) and high specificity (avoiding false positives) is often challenging. Biomarkers that are highly sensitive may yield false-positive results, while highly specific biomarkers may miss some cases of cancer.

Biomarker development requires rigorous validation across large and diverse patient populations to ensure their reliability and reproducibility. This validation process can be time-consuming and costly. The development of robust and reliable laboratory techniques for detecting and measuring biomarkers is critical. Standardizing these techniques across different laboratories and platforms can be challenging, affecting the consistency and comparability of results. The development and implementation of biomarkers for cancer diagnosis involve ethical considerations, such as ensuring patient privacy, obtaining informed consent, and addressing issues of equity and access to testing. Regulatory approval processes and adherence to quality control standards also play crucial roles in biomarker development.

The availability and cost of biomarker tests can limit their widespread adoption. Some biomarker tests may require specialized equipment or expertise, making them less accessible, particularly in resource-limited settings. Biomarkers for early cancer detection should ideally allow for longitudinal monitoring to track disease progression, treatment response, and recurrence. Developing biomarkers that provide real-time and dynamic information about the disease can be challenging.

Thus, along with the undisputable advantages, the application of biomarkers for early cancer diagnosis has also certain disadvantages (Table 6). It is important to note that the advantages and disadvantages of biomarkers can vary depending on the specific biomarker, cancer type, and stage of cancer. Each biomarker should be evaluated and interpreted in the context of clinical guidelines and in collaboration with healthcare professionals.

**Table 6:** Advantages and Disadvantages of the Biomarkers for Early Cancer Detection

**Advantages**	**Disadvantages**
**Early Detection:** Biomarkers provide a means to detect cancer at an early stage, when treatment is more likely to be successful. Early diagnosis can lead to improved patient outcomes and increased survival rates.	**Lack of Specificity:** Many biomarkers may be elevated in non-cancerous conditions, leading to false-positive results. This can result in unnecessary additional testing, procedures, and anxiety for patients.
**Non-invasive or Minimally Invasive:** Many biomarker tests involve simple blood tests or urine tests, making them non-invasive or minimally invasive procedures. This reduces patient discomfort and the need for invasive diagnostic procedures like biopsies.	**False Negatives:** Biomarkers may not be present or detectable in all individuals with cancer. False-negative results can lead to delayed diagnosis and missed opportunities for early intervention.
**Monitoring Treatment Response:** Biomarkers can be used to monitor the response to cancer treatments, such as chemotherapy or targeted therapies. They provide a way to assess treatment effectiveness and make necessary adjustments to the treatment plan.	**Variability and Standardization:** Biomarker levels can vary among individuals, making it challenging to establish universal cutoff values for diagnosis or risk assessment. Standardizing biomarker measurement techniques across different laboratories and platforms is essential for consistent and reliable results.
**Personalized Medicine:** Biomarkers have the potential to guide personalized treatment approaches. By identifying specific biomarkers associated with certain cancers, healthcare professionals can tailor treatment strategies to individual patients, leading to more effective and targeted therapies.	**Limited Sensitivity:** Some biomarkers may not have high sensitivity in the early stages of cancer or for certain types of cancer. This can result in missed diagnoses or delayed detection.
**Risk Assessment and Screening:** Biomarkers can assist in identifying individuals at higher risk of developing certain cancers. This enables targeted screening programs and preventive interventions for individuals at elevated risk, contributing to early detection and prevention.	**Cost and Accessibility:** Biomarker testing may involve costs including laboratory testing fees and specialized equipment. The availability and accessibility of biomarker tests can be limited, particularly in resource-limited settings, which may impact widespread adoption and equitable access to early cancer diagnosis.
**Prognostic Indicators:** Some biomarkers can provide valuable prognostic information, helping to predict disease progression and overall patient prognosis. This information can guide treatment decisions and assist in patient counseling and support.	**Ethical and Regulatory Considerations:** The development and implementation of biomarkers raise ethical considerations, such as ensuring patient privacy, obtaining informed consent, and addressing issues of equity and access to testing. Regulatory approval processes and adherence to quality control standards are important for the reliable and safe use of biomarkers.

Despite all challenges and roadblocks, the field of cancer biomarker research continues to advance, with ongoing efforts to discover and validate new biomarkers. As technology improves and our understanding of cancer biology deepens, the potential for earlier and more accurate cancer diagnosis becomes increasingly achievable. These efforts have the potential to significantly reduce cancer-related morbidity and mortality in the future.

## Supporting Information Available

Supplemental Table S1: Selected markers based on Validity and Disease Diagnosis score, a table of biomarkers in liver or pancreatic cancer that can be measured through specimens obtained non-invasively vs the validity and disease diagnosis score for each of the biomarkers, as well as their association with other clinical outcomes, whenever available (PDF)

## Abbreviations

mRNA: messenger RNA
ncRNA: non-coding RNA
lncRNA: long non-coding RNA
miRNA: microRNA
BRCA1/2: breast cancer gene 1/2
cfDNA: cell-free DNA
ctDNA: circulating tumor DNA
MRI: magnetic resonance imaging
CT: computed tomography
PET: positron emission tomography
ELISA: enzyme-linked immunosorbent assay
PCR: polymerase chain reaction
SPR: surface plasmon resonance
SERS: surface-enhanced Raman spectroscopy
AFP: alpha-fetoprotein
PSA: prostate-specific antigen
CEA: carcinoembryonic antigen
CA19-9: cancer antigen 19-9
CA72-4: cancer antigen 72-4
CA125: cancer antigen 125
CA15-3: cancer antigen 15-3
TCGA: The Cancer Genome Atlas
CTC: circulating tumor cell
HER2: human epidermal growth factor receptor-2
EUS: endoscopic ultrasound
FNA: fine-needle aspiration
HCC: hepatocellular carcinoma
MCED: multi-cancer early detection
FDA: U.S. Food and Drug Administration
AI: artificial intelligence
ML: machine learning
VOC: volatile organic compound
