# Supplementary material for: Biomarkers for Early Cancer Detection: A Landscape View of Recent Advancements, Spotlighting Pancreatic and Liver Cancers
Source: ACS Pharmacol Transl Sci. 2024 Feb 14;7(3):586–613. doi: 10.1021/acsptsci.3c00346 (PMC10928905; doi:10.1021/acsptsci.3c00346)
Supplement: Supplementary file 1 — pt3c00346_si_001.pdf [file pt3c00346_si_001.pdf]

Supporting Information:

Biomarkers for early cancer detection: A landscape view of recent advancements, spotlighting pancreatic and liver cancers

Rumiana Tenchov†[1], Aparna K. Sapra†*[2], Janet Sasso[1], Krittika Ralhan[3], Anusha Tummala[2], Norman Azoulay[2], Qiongqiong Angela Zhou*[1]

[1]CAS, a division of the American Chemical Society, Columbus OH 43210, USA

[2]Excelra Knowledge Solutions Pvt. Ltd., Hyderabad-500039, India

[3]ACS International India Pvt. Ltd., Pune-411044, India

† Authors R.T. and A.K.S. contributed equally to this paper

*Corresponding authors: qzhou@cas.org

aparna.sapra@excelra.com

Supplemental Table S1. Selected markers based on Validity and Disease Diagnosis score

Table of biomarkers in liver or pancreatic cancer that can be measured through specimens obtained non-invasively vs. the validity and disease diagnosis score for each of the biomarkers, as well as their association with other clinical outcomes, whenever available.

DISEASE	SPECIMEN	BIOMARKER	VALIDITY SCORE	DISEASE DIAGNOSIS SCORE	Other associated outcomes
Liver Cancer	Blood|Breath Sample|Plasma|Serum|Urine	ALPHA FETOPROTEIN (AFP)	815	152	Overall survival; Progression free survival (LC); Metastasis (LC)
Pancreatic Cancer	Blood|Plasma|Serum	CARBOHYDRATE ANTIGEN 19-9 (CA19-9)	419	41	Overall survival (LC+PC); Metastasis (LC); Lymph node metastasis (LC)
Liver Cancer	Blood|Plasma|Serum	GLUTAMIC-OXALOACETIC TRANSAMINASE 1 (GOT1)	233	36	Overall survival (LC)
Liver Cancer	Blood|Plasma|Serum	GLUTAMIC--PYRUVIC TRANSAMINASE (GPT)	229	33	Overall survival (LC)
Liver Cancer	Blood|Plasma|Serum	ALBUMIN (ALB)	227	-31	Overall survival (LC+PC)
Liver Cancer	Blood|Plasma|Serum	BILIRUBIN	197	23	Overall survival (LC)
Liver Cancer	Blood|Plasma|Serum	PLATELETS	155	-16	0
Liver Cancer	Blood|Plasma|Serum	DES-GAMMA CARBOXYPROTHROMBIN (DCP)	113	29	Overall survival (LC)
Pancreatic Cancer	Blood|Plasma|Serum	CARCINOEMBRYONIC ANTIGEN (CEA)	97	12	Overall survival (PC)
Liver Cancer	Blood|PBMC|Plasma|Serum	NEUTROPHIL TO LYMPHOCYTE RATIO (NLR)	93	9	Overall survival (LC+PC); Progression free survival (LC)
Liver Cancer	Blood|Serum	GAMMA-GLUTAMYL TRANSPEPTIDASE (GGT)	73	15	Overall survival (LC)
Liver Cancer	Blood|Plasma|Serum	ALKALINE PHOSPHATASE (ALP)	70	17	Overall survival (LC)
Pancreatic Cancer	Blood|Plasma|Serum	CD274 MOLECULE (CD274)	62	8	Overall survival (LC+PC); Immune cell infiltration (LC)
Liver Cancer	Blood|Plasma|Serum	HBV DNA	55	8	Overall survival (LC)
Liver Cancer	Blood|Plasma|Serum	CREATININE	52	6	0
Liver Cancer	Blood|PBMC|Serum	CD8+ T CELLS	52	-5	Overall survival (LC)
Liver Cancer	Blood|Plasma|Serum	CD274 MOLECULE (CD274)	48	8	Overall survival (LC+PC); Immune cell infiltration (LC)
Liver Cancer	Blood|Serum	C-REACTIVE PROTEIN (CRP)	47	6	Overall survival (LC+PC)
Pancreatic Cancer	Blood|Plasma|Serum	KRAS VARIANT	44	5	Overall survival (PC)
Pancreatic Cancer	Blood|Plasma|Serum	INTERLEUKIN 6 (IL6)	43	11	Overall survival (LC)
Liver Cancer	Blood|Plasma|Serum	HEMOGLOBIN	39	-5	Overall survival (LC)
Pancreatic Cancer	Blood|Plasma|Serum	BILIRUBIN	33	5	Overall survival (LC)
Pancreatic Cancer	Blood|Plasma|Serum	MUCIN 16, CELL SURFACE ASSOCIATED (MUC16)	33	5	0

Liver Cancer	Blood|Plasma|Serum	VASCULAR ENDOTHELIAL GROWTH FACTOR (VEGF)	31	5	0
Liver Cancer	Blood|Plasma|Serum	CARBOHYDRATE ANTIGEN 19-9 (CA19-9)	29	5	Overall survival (LC+PC); Metastasis (LC); Lymph node metastasis (LC)
Liver Cancer	Blood|Serum	LENS CULINARIS AGGLUTININ A-REACTIVE FRACTION OF AFP (AFP-L3)	28	8	0
Liver Cancer	Blood|Serum	CADHERIN 1 (CDH1)	26	-7	0
Liver Cancer	Blood|Plasma|Serum	GLYPICAN 3 (GPC3)	26	18	0
Liver Cancer	Plasma|Serum	SECRETED PHOSPHOPROTEIN 1 (SPP1)	25	13	Overall survival (LC)
Pancreatic Cancer	Blood|Plasma|Serum	C-X-C MOTIF CHEMOKINE LIGAND 8 (CXCL8)	23	7	Overall survival (LC)
Liver Cancer	Blood	HYPOXIA INDUCIBLE FACTOR 1 SUBUNIT ALPHA (HIF1A)	21	11	Overall survival (LC)
Liver Cancer	Blood	TUMOR PROTEIN P53 (TP53)	21	6	0
Liver Cancer	Blood|Plasma|Serum	CARCINOEMBRYONIC ANTIGEN (CEA)	21	6	Overall survival (PC)
Liver Cancer	Blood|Serum	CHOLESTEROL	20	-7	0
Liver Cancer	Serum	CATENIN BETA 1 (CTNNB1)	20	9	Overall survival (LC)
Liver Cancer	Serum	MYC PROTO-ONCOGENE, BHLH TRANSCRIPTION FACTOR (MYC)	20	13	0
Liver Cancer	Blood|Serum	GOLGI MEMBRANE PROTEIN 1 (GOLM1)	18	10	#N/A
Pancreatic Cancer	Serum	MET PROTO-ONCOGENE, RECEPTOR TYROSINE KINASE (MET)	17	5	#N/A
Pancreatic Cancer	Blood|Plasma|Serum	INTERLEUKIN 1 BETA (IL1B)	17	5	#N/A
Liver Cancer	Blood|serum	VASCULAR ENDOTHELIAL GROWTH FACTOR A (VEGFA)	17	7	#N/A
Liver Cancer	Plasma	MET PROTO-ONCOGENE, RECEPTOR TYROSINE KINASE (MET)	17	10	#N/A
Pancreatic Cancer	Serum	HYPOXIA INDUCIBLE FACTOR 1 SUBUNIT ALPHA (HIF1A)	17	5	Overall survival (LC)
Liver Cancer	Blood	POLO LIKE KINASE 1 (PLK1)	16	7	#N/A
Liver Cancer	Plasma	VIMENTIN (VIM)	16	8	#N/A
Liver Cancer	Blood|PBMC|Serum	REGULATORY T CELLS	15	8	#N/A
Liver Cancer	PBMC	SIGNAL TRANSDUCER AND ACTIVATOR OF TRANSCRIPTION 3 (STAT3)	14	5	#N/A
Liver Cancer	Blood|Serum	TRIGLYCERIDE	14	-5	#N/A

Liver Cancer	Blood	AKT SERINE/THREONINE KINASE 1 (AKT1)	14	7	#N/A
Liver Cancer	Blood	METASTASIS ASSOCIATED LUNG ADENOCARCINOMA TRANSCRIPT 1 (MALAT1)	14	9	#N/A
Liver Cancer	Blood	CYCLIN DEPENDENT KINASE 4 (CDK4)	14	7	#N/A
Liver Cancer	Blood	SMALL NUCLEOLAR RNA HOST GENE 1 (SNHG1)	13	6	#N/A
Liver Cancer	Blood|Plasma	CYTOSKELETON REGULATOR RNA (CYTOR)	13	7	#N/A
Liver Cancer	Blood|Serum	HSA-MIR-139-5P	12	-5	#N/A
Liver Cancer	Blood|Plasma|Serum	ALPHA FETOPROTEIN (AFP)|DES-GAMMA CARBOXYPROTHROMBIN (DCP)	11	7	#N/A
Liver Cancer	Serum	NUCLEAR PARASPECKLE ASSEMBLY TRANSCRIPT 1 (NEAT1)	11	6	#N/A
Liver Cancer	Blood|Serum	HSA-MIR-221	10	7	#N/A
Pancreatic Cancer	Plasma	5'-NUCLEOTIDASE ECTO (NT5E)	10	5	#N/A
Liver Cancer	Serum	COLORECTAL NEOPLASIA DIFFERENTIALLY EXPRESSED (CRNDE)	10	8	#N/A
Liver Cancer	Serum	HEXOKINASE 2 (HK2)	10	5	#N/A
Liver Cancer	Blood	HSA-MIR-199A-3P	10	-5	#N/A
Liver Cancer	Blood|Serum	HSA-MIR-34A	10	-6	#N/A
Liver Cancer	Serum	PARAOXONASE 1 (PON1)	10	-6	#N/A
Liver Cancer	Serum	ANNEXIN A2 (ANXA2)	9	5	#N/A
Liver Cancer	Serum	GLUCOSE TRANSPORTER 1 (GLUT1)	9	5	#N/A
Liver Cancer	Serum	HSA-MIR-145	9	-6	#N/A
Liver Cancer	Blood|Serum	DES-GAMMA CARBOXYPROTHROMBIN (DCP)|ALPHA FETOPROTEIN (AFP)	9	7	#N/A
Pancreatic Cancer	Serum	LAMININ SUBUNIT GAMMA 2 (LAMC2)	8	5	#N/A
Liver Cancer	Blood|Serum	MATRIX METALLOPEPTIDASE 2 (MMP2)	8	5	#N/A
Liver Cancer	Blood|Plasma|Serum	HEPATOCELLULAR CARCINOMA UP-REGULATED LONG NON-CODING RNA (HULC)	8	5	#N/A
Liver Cancer	Serum	HSA-MIR-199A-5P	8	-6	#N/A
Liver Cancer	Serum	MINICHROMOSOME MAINTENANCE COMPLEX COMPONENT 6 (MCM6)	8	6	#N/A

Pancreatic Cancer	Blood|Plasma	THROMBOSPONDIN 2 (THBS2)	8	6	#N/A
Liver Cancer	Serum	TYROSINE 3-MONOOXYGENASE/TRYPTOPHAN 5-MONOOXYGENASE ACTIVATION PROTEIN ZETA (YWHAZ)	8	5	#N/A
Liver Cancer	Serum	ALDO-KETO REDUCTASE FAMILY 1 MEMBER B10 (AKR1B10)	7	5	#N/A
Liver Cancer	Blood|Serum	GROWTH DIFFERENTIATION FACTOR 15 (GDF15)	7	5	#N/A
Pancreatic Cancer	Blood|Plasma	HSA-MIR-155	7	5	#N/A
Liver Cancer	Plasma	HSA-MIR-192	7	-5	#N/A
Pancreatic Cancer	Blood|Plasma|Serum	HSA-MIR-21	6	6	#N/A
